# Improving the Usability of Donor Data from Registries Using an Extreme Learning Machine Approach to Upgrade Low-/Mid- to High-Resolution HLA Data

**DOI:** 10.3390/genes17101239

**Published:** 2026-10-08

**Authors:** Stavros Kepentzis, Theofanis Chatzistamatiou, Jason Digalakis, Ourania Petropoulou, George K. Matsopoulos, Dimitrios Koutsouris

**Affiliations:** 1 School of Electrical and Computer Engineering, National Technical University of Athens, 9 Iroon Polytechniou St., Zografou Polytechnic Campus, 15772 Athens, Greece; skepenjis@biomed.ntua.gr (S.K.); dkoutsou@biomed.ntua.gr (D.K.); 2 Clinical Biochemistry Laboratory, “Sismanoglio” General Hospital of Attica, 1 Sismanogliou St., 15126 Marousi, Greece

**Keywords:** HLA typing, extreme learning machine, imputation, donor registry, hematopoietic stem cell transplantation, machine learning, histocompatibility

## Abstract

Background/Objectives: High-resolution Human Leukocyte Antigen (HLA) typing is required for unrelated donor selection, yet many registry records remain low- or intermediate-resolution. Haplotype frequency imputation is the standard remedy, but its accuracy decreases for rare haplotypes. Methods: A locus-specific Extreme Learning Machine (ELM) framework, trained on the Greek national donor registry (*n* = 117,345), upgrades low-/intermediate-resolution genotypes and imputes untyped loci; output weights are solved analytically. It was validated on two independent Greek cohorts (*n* = 20,100; 4353) against GRIMM and two expectation maximization baselines, on the same donors. Results: Accuracy depended on the high-resolution context available (94% for HLA-A with four anchor loci; 19–22% with none) and on the posterior probability threshold (78–91% ORAM, 70–84% GRPT), while call rate fell from 98.1% to ~41%; the two cannot be maximized together. GRIMM called fewer donors (1.8–47.9%) at comparable or higher accuracy. Allele-vocabulary abridgment excluded 14–47% of external-cohort allele calls; stratified by vocabulary membership, ORAM accuracy was 94.2% for in-vocabulary donors and 73.7% for the rest. The training procedure has no convergence guarantee. Conclusions: ELM upgrading converts legacy registry data into probabilistic high-resolution calls, with registry-scale training completing in minutes. These calls can narrow donor searches but cannot substitute for confirmatory typing.

## 1. Introduction

Matching donors and recipients at the Human Leukocyte Antigen (HLA) loci remains the single most important genetic determinant of outcome in unrelated hematopoietic stem cell transplantation (HSCT) [1], and contemporary matching algorithms increasingly rely on allele-level, four-digit (or higher)-resolution typing [2,3,4,5]. However, the majority of volunteer donors enrolled in international registries over the past two decades were typed using serologic or intermediate-resolution DNA-based methods that were adequate for the technology and clinical practice of their era yet insufficient for present-day matching algorithms [6].

This creates a large and growing gap between the resolution at which donor HLA data were originally captured and the resolution now required for optimal donor selection. Re-typing thousands of archived donor samples is not economically or logistically feasible for most registries. Statistical imputation has therefore become the pragmatic solution: low- or intermediate-resolution genotypes are converted into probable high-resolution haplotype pairs using reference haplotype frequency tables built from previously confirmed high-resolution typings [7], an approach exemplified by tools such as the National Marrow Donor Program (NMDP) HaploStats service [8,9,10], graph-based methods like GRIMM [11], and related tools, including Hapl-o-Mat [12], HIBAG [13], and HLA*IMP [14].

Haplotype frequency imputation performs well for common haplotypes in well-represented populations but is known to decrease in accuracy for rare haplotypes, for loci with extreme polymorphism, and for individuals from ancestries that are sparsely represented in the reference panel, specifically populations for whom improving registry usability matters most [15,16]. In addition, haplotype frequency tables must be periodically recomputed as new high-resolution data accrue, which is a computationally intensive process at registry scale [7].

Machine learning offers an alternative or complementary route to imputation. Supervised classifiers trained on paired low-/mid- and high-resolution typings can, in principle, learn population-specific patterns of linkage disequilibrium directly from the data without requiring an explicit haplotype frequency model. Neural network approaches, however, tend to not be preferred for this task because iterative gradient-based training [17] is slow and must be repeated whenever new confirmatory typing becomes available, which is a near-continuous occurrence in an active registry.

The Extreme Learning Machine (ELM) is a training algorithm for single-hidden-layer feedforward networks in which the input-to-hidden weights and biases are assigned randomly and fixed, while the hidden-to-output weights are solved analytically in closed form using the Moore–Penrose generalized inverse, avoiding iterative back-propagation entirely [18,19,20,21]. This enables ELM training times that are typically orders of magnitude faster than conventional neural networks while retaining competitive generalization performance, a combination that is attractive for a registry setting where the model may need to be retrained frequently as new high-resolution reference data accumulate.

It is important to distinguish two related but distinct tasks that a framework of this kind can address, both of which we evaluate below. The first is resolution upgrading in the strict sense: a locus has been typed, but only at low or intermediate resolution, and the task is to predict the corresponding high-resolution allele(s). The second is missing-locus imputation: a locus was not typed at all, and the task is to infer plausible high-resolution alleles for that locus using linkage disequilibrium with loci that were typed. These tasks differ in statistical difficulty, since missing-locus imputation discards the (weak but non-zero) information content of a low-resolution call at the target locus itself, and we report results for both rather than treat them as a single undifferentiated ‘imputation accuracy’. Resolution upgrading results are tabulated in Section 3.1, and missing-locus imputation is evaluated against a haplotype frequency baseline in Supplementary Section S4.1.

In this article, we describe the development and benchmarking of an ELM-based framework to upgrade low-/mid-resolution HLA genotypes to probabilistic high-resolution calls and to impute entirely untyped loci at the loci most relevant to unrelated donor selection. We applied the proposed approach to a national donor registry and compared it, on the same donors, against GRIMM and two other haplotype frequency baselines. We report the accuracy and call rate jointly rather than as independent maxima; accuracy and computational cost against conventional machine learning benchmarks are characterized separately on a simulated reference dataset (Supplementary Section S3).

## 2. Materials and Methods

### 2.1. Reference Cohort and Data Sources

The reference cohort consisted of donor records held by the Hellenic Transplant Organization (HTO) national donor registry, established in 2002 through recruitment at five donor centers between 2002 and 2019 (*n* = 117,345). Two other cohorts, external to the HTO training registry and used exclusively for validation, are referenced throughout this article by abbreviation: ORAM (Orama Elpidas Donor Center; 20,100 individuals) and GRPT (Evangelismos Hospital Donor Center; 4353 individuals). Both are detailed below. As a population-based national registry, the dataset reflects the demographic and geographic composition of donor recruitment in Greece over that period; we do not characterize it as free of bias, since selection effects related to recruitment site, timing, and donor demographics cannot be excluded and were not formally quantified in this study. Personal identification fields (registry and center identifiers) were removed prior to analysis; columns with less than 5% completeness were dropped, rows with 100% completeness were retained, and records containing non-numeric/missing date fields were excluded. Approximately 20% of the registry consisted of records held only at low resolution (Table 1); the remaining records were held at intermediate or high resolution.

Two other cohorts, external to the HTO training registry, were used exclusively for validation and were not used in training or hyperparameter selection: ORAM and GRPT, both of Greek ancestry. A locus-level audit of each cohort’s raw typing file (Table 1) shows that neither cohort is uniformly typed at high (second-field) resolution across all five loci: the large majority of donors in both cohorts are typed only at intermediate (ambiguous, two-field) resolution at HLA-A, -B, and -DRB1 (83.6–99.8% of donors, depending on locus and cohort), and the majority are untyped at HLA-C and HLA-DQB1 (77.0–100% of donors); only a minority of donors per locus (7.9–36.1% in ORAM, 0.2–1.6% in GRPT) have unambiguous, high (second-field)-resolution typing. It is specifically this high-resolution minority, per locus, that provides confirmatory two-field ground truth against which upgraded/imputed calls were scored in Section 3.1, Section 3.2 and Section 3.3 and Supplementary Section S4; donors typed at only intermediate resolution or untyped at a given locus contribute to that locus’s input data but not to its accuracy scoring. ORAM and GRPT are administratively distinct from the five HTO donor centers. We additionally performed a record-level deduplication check on full donor identifiers (registry ID including center code, not just the numeric portion) across the HTO training registry, ORAM, and GRPT; the verified files used throughout this article share zero donor identifiers pairwise (0/0/0 across all three comparisons), confirming donor-level independence of the two external validation cohorts from the training registry.

### 2.2. Definition of Resolution Levels and Handling of Ambiguity

Table 2 summarizes the resolution categories used throughout this work, with explicit examples. Allele designations and field depth follow IPD-IMGT/HLA nomenclature throughout [22,23].

A genotype is ambiguous when the low-/mid-resolution typing is compatible with more than one high-resolution allele (or, at the level of a full multilocus genotype, with more than one diplotype). Ambiguous genotypes were not collapsed into a single composite (‘multiallelic’) code; instead, each was expanded into the full set of high-resolution diplotypes consistent with the observed typing, and the algorithm assigns a probability to every member of that compatible set rather than committing to one (Section 2.4 and Section 2.5). Where a locus was entirely untyped (recorded as unknown rather than typed-but-ambiguous), all alleles observed at that locus in the reference population were treated as possible and enumerated in the same way; this is the missing-locus imputation task, which was distinguished from resolution upgrading in Section 1. Consistent with common registry practice, models were trained to predict two-field, unambiguous high-resolution alleles (e.g., A*02:01) as the primary target, with a secondary evaluation at the three-field level where sequencing depth allowed.

### 2.3. Definition of Accuracy, Call Rate, and Posterior Probability

We distinguish three related but separate quantifiable criteria that are reported throughout the Results, with two other measures defined below. Per-locus accuracy is the proportion of individuals for whom both predicted high-resolution alleles at a given locus exactly matched the confirmatory high-resolution typing at that locus. Overall (multilocus) accuracy is the corresponding proportion computed jointly across all loci evaluated in a given experiment, i.e., those requiring an exact match at every predicted locus simultaneously. Unless stated otherwise, the accuracy figures reported per locus in Section 3.1 are single-locus accuracies, not the (necessarily lower) joint multilocus accuracy, and the two should not be read interchangeably. Call rate is the proportion of input genotypes for which the algorithm returns a prediction at or above a specified confidence threshold, out of all genotypes submitted; a genotype can fail to receive a call either because no compatible diplotype was found in the reference haplotype database or because no compatible diplotype reached the requested posterior probability threshold.

For a genotype with n diplotypes compatible with the observed (possibly ambiguous or incomplete) typing, each compatible diplotype dt is assigned a likelihood *L*(*dt*) computed from reference haplotype frequencies *f* under Hardy–Weinberg proportions [24]: for a homozygous diplotype, *L* = *f*^2^; for a heterozygous diplotype composed of haplotypes *h*1 and *h*2, *L* = 2·*f*(*h*1)·*f*(*h*2). The posterior probability of a given diplotype is then its likelihood normalized by the summed likelihood of all *n* compatible diplotypes, *Post-P*(*dt*) = *L*(*dt*)/*Σ_i_L*(*d_i_*), and the posterior probability reported for the top prediction is *Post-P* = *max_i_*(*L(d_i_*))/*Σ_i_L*(*d_i_*). A confidence threshold on *Post-P* can be applied to accept or withhold a call. This confidence threshold is a call acceptance criterion applied by the model at inference time, distinct from the 95% confidence intervals reported throughout this article (Section 2.7), which instead quantify the statistical uncertainty of an already-computed accuracy or call rate estimate; the two are not directly related. Concretely, we report results across the full range of this threshold (0% to 82% posterior probability; Section 3.1) rather than commit to one fixed operating point in the headline results, since raising the threshold increases the accuracy of returned calls (since low-confidence, more error-prone predictions are withheld) but also consequently decreases the call rate (since fewer genotypes clear the bar). Because these two metrics move in opposite directions along the same threshold, they should always be reported together, at a stated threshold or as a joint curve, rather than as independent headline numbers; the empirical threshold curve for this registry is reported in Section 3.1, not here, since it is a result rather than a methodological definition.

Because this article reports several related performance measures without always restating their definitions at the point of use, Table 3 lists them in one place for reference.

### 2.4. Network Architecture and Input/Output Representation

We first clarify how randomness, which is the defining feature of the base ELM, coexists with the biologically structured, multilayer network used here, since these can appear contradictory if not explicitly reconciled. The network’s topology, i.e., which nodes exist and which nodes are permitted to connect to which, is fixed in advance based on domain knowledge of the HLA locus structure and is not learned: input-layer nodes correspond to observed alleles/genotype fragments; first-hidden-layer (HL-1) nodes correspond to partial haplotypes spanning three loci; second-hidden-layer (HL-2) nodes correspond to partial haplotypes spanning five loci; the third hidden layer (HL-3) represents complete, fully enumerated haplotypes; and output-layer nodes represent candidate diplotypes. An input node is permitted to connect only to HL-1 nodes whose partial haplotype contains one of the alleles that input node represents, an HL-1 node only to HL-2 nodes whose broader partial haplotype is a superset of it, and so forth. What is randomly generated and left untrained, consistent with the base ELM formulation, is the set of connection weights and biases within this fixed topology, not the topology itself; the output-layer (hidden-to-output) weights are then the only parameters solved analytically, through the Moore–Penrose generalized inverse, exactly as in a conventional ELM. In other words, this is a domain-structured (topology-constrained) ELM: population genetic knowledge is leveraged to determine which weighted connections can exist, while the ELM algorithm determines their values.

For a diploid genotype spanning x loci, the number of theoretically possible diplotypes is *N* = 2^(*x*−1)^ (e.g., four diplotypes for a three-locus heterozygous genotype); diplotypes whose corresponding haplotype has never been observed in the reference database are removed from consideration, reducing N in practice. Because the allele vocabulary was abridged to the minimum set required to describe the training cohort (to bound the size of the enumeration and the resulting network), this abridgment step preferentially removes very rare alleles and can bias predictions toward common alleles. We first verified that ORAM (*n* = 20,100) and GRPT (*n* = 4353) share no donor identifier with the HTO training registry or with each other (0/0/0 exact-match donor IDs across all three pairwise comparisons; coincidental genotype matches, expected under a shared national allele frequency spectrum, were 4.6% and 1.5%, respectively), and we then measured what fraction of ORAM/GRPT allele calls fall outside the HTO-derived 99–coverage vocabulary (Table 4, Figure S1): 18–47% of ORAM allele calls and, excluding HLA-DQB1 (0.0%, near-untyped in GRPT; Section 2.1), 14–22% of the small number of unambiguously typed GRPT calls fall outside the training vocabulary, depending on locus. For comparison, we separately quantified this same abridgment bias within the HTO training registry itself (117,345 donor records across five donor centers, unambiguous two-field DNA typing), asking per locus how many distinct alleles a vocabulary must retain to cover a given proportion of observed allele calls (Table 5, Figure S2). At every locus, the great majority of distinct alleles observed are unique: 18–32% of distinct alleles at each locus were observed only once in the entire registry. A vocabulary retaining the alleles needed to cover 99% of allele calls excludes 58–81% of the distinct alleles observed per locus, yet leaves out only 0.4–0.9% of allele calls, confirming that abridgment discards diversity disproportionately from the low-frequency tail compared to commonly typed alleles. At the donor level, restricting to the 86,002 donors (73.3% of the registry) with all five loci typed at unambiguous two-field resolution, 6.9% carried at least one allele outside the 99–coverage vocabulary at one or more loci, i.e., a small but non-trivial minority of donors whose true allele would not be representable by an abridged model at that coverage level. The cross-cohort exclusion rate (Table 4) is substantially higher than this within-HTO self-consistency figure (Table 5), indicating that the HTO-trained vocabulary generalizes markedly less completely to the external cohorts than the within-registry rarity analysis alone would suggest; we treat this as a material limitation on the abridgment step rather than a fully resolved one (see the Section 5).

**Figure 1:**
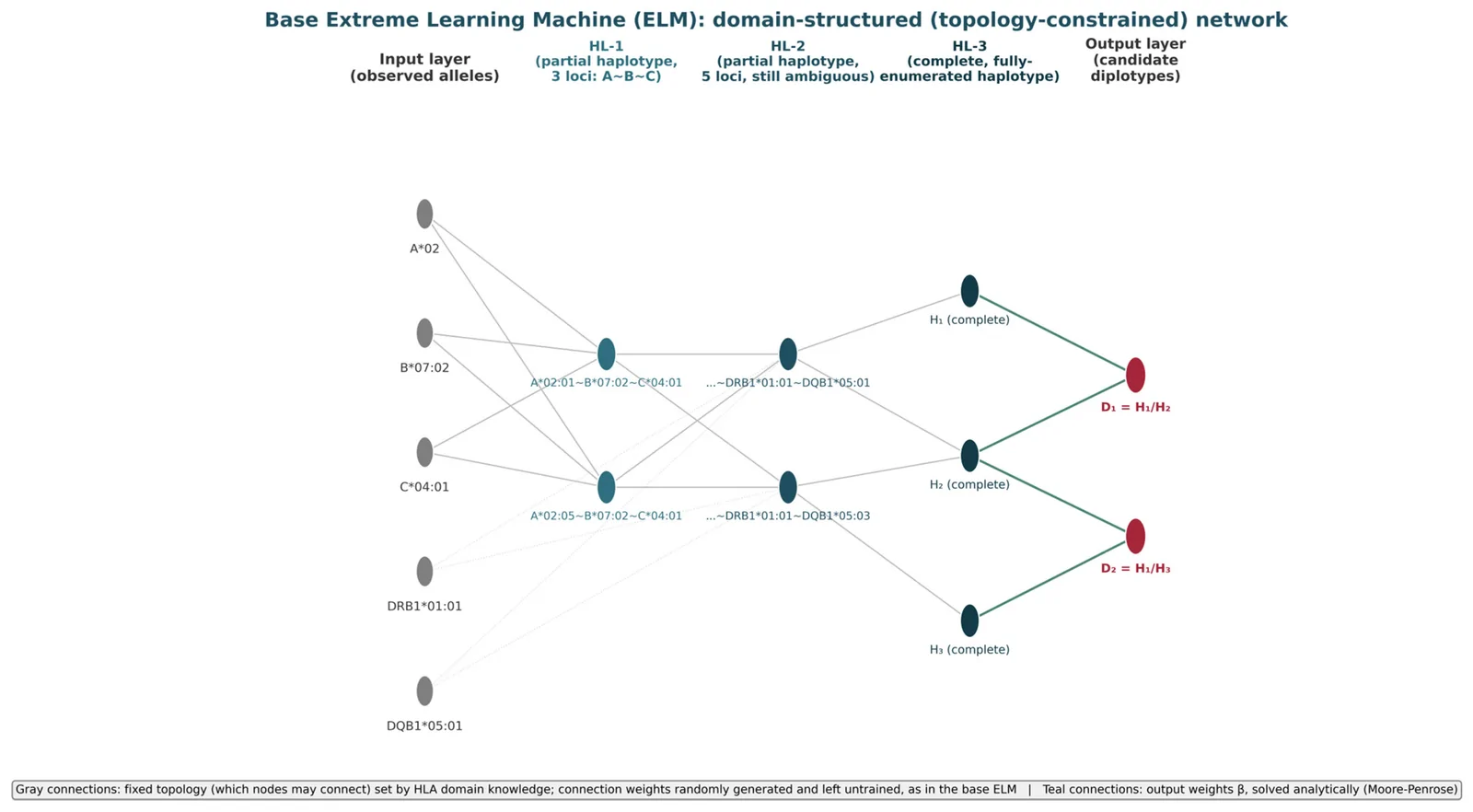
Schematic of the domain-structured (topology-constrained) base Extreme Learning Machine (ELM) used as the algorithmic core of the framework, illustrated with the worked example introduced in the text (input alleles A*02, B*07:02, C*04:01, DRB1*01:01, DQB1*05:01). Grey nodes are observed input alleles, teal nodes the structured hidden layers HL-1 to HL-3, and dark red nodes the candidate output diplotypes; grey connections carry the fixed topology with untrained random weights, and teal connections the analytically solved output weights. The asterisk in allele names (for example, A*02:01) is part of standard HLA nomenclature and is not a footnote marker. Input-layer nodes correspond to observed alleles; the hidden layer is organized into three hierarchical levels reflecting progressively more complete partial haplotypes—HL-1 (three loci), HL-2 (five loci, still ambiguous), and HL-3 (complete, fully enumerated haplotype)—linked by a sparse, topology-constrained connectivity pattern that follows allele containment logic. Input-to-hidden weights and biases along these connections are randomly generated and left untrained (gray connections), while output-layer nodes represent candidate diplotypes, each formed by pairing two HL-3 haplotype nodes, and hidden-to-output weights β are solved analytically in closed form through the Moore–Penrose pseudoinverse (teal connections).

Input encoding, output decoding, and a worked example. An input genotype is represented by activating the input-layer nodes corresponding to each observed (possibly ambiguous or missing) locus call; for a missing locus, all alleles observed at that locus in the reference population are treated as simultaneously present at the input layer, each with its own downstream connections. Forward propagation activates every HL-1, HL-2, and HL-3 node reachable from the activated input nodes under the fixed topology described above, and every output node (candidate diplotype) reachable from an activated HL-3 node receives a weighted signal. The predicted output is not a single deterministic label but the ranked list of activated output nodes together with their posterior probabilities (Section 2.3). The network’s output weights enter this step directly: the score h(g)·β of each candidate diplotype determines which output nodes are retained—those with a positive score—and in what order, and the posterior probability of Section 2.3 is then normalized over that retained set rather than over every diplotype compatible with the genotype (Algorithm S2). A donor for whom no candidate attains a positive score yields no call. This is what distinguishes the framework from posterior scoring against a fixed haplotype frequency table; a single ‘top-1’ high-resolution diplotype call is obtained by taking the argmax of this list, and a ‘top-k’ call by taking its k highest-probability members. As a concrete example, a donor typed as HLA-A*02 (low resolution) with HLA-B, -C, -DRB1, and -DQB1 previously confirmed at high resolution activates the A*02 input node and the four known-locus input nodes; this activates every HL-1/HL-2/HL-3 node consistent with a haplotype containing A*02 and the four known high-resolution alleles, and the output layer returns the compatible high-resolution A-locus alleles (e.g., A*02:01, A*02:05) ranked by posterior probability, from which the top-1 or top-k call is read off. Figure 1 illustrates this domain-structured architecture and the worked example schematically.

### 2.5. Training Procedure

Because the true distribution of the haplotype population is unknown, the network cannot be trained against experimentally verified ground-truth haplotype labels for every training genotype; instead, training proceeds iteratively against the distribution of diplotypes consistent with each observed (possibly ambiguous) genotype. At each training step, a genotype is drawn without replacement from the training population, and a target diplotype is drawn from the set of diplotypes compatible with that genotype under an initial assumption that all compatible diplotypes are equally probable. The genotype is presented to the input layer and propagated forward to obtain one or more candidate diplotype classifications; the Hamming distance between each candidate and the sampled target diplotype defines the pattern error for that step. Hidden-layer parameters follow the standard ELM formulation and are randomly assigned and left untuned; output-layer weights β are updated through the closed-form least squares solution (Moore–Penrose pseudoinverse) given the accumulated hidden-layer output matrix H and target matrix T. H and T accumulate across epochs and are not reset at the start of each one: every epoch draws a fresh target diplotype for each genotype from the uniform prior over its compatible set and appends it, so β at epoch k is the least squares solution over the union of all samples drawn up to that point, and the sampled-target distribution approaches the prior-weighted marginal as k increases (Algorithm S1). Sampling continues until the training population is exhausted, at which point the population is reshuffled and another epoch begins, or training stops once total error falls below a predetermined threshold. Across the 125 per-locus training runs reported here (five loci × 25 repetitions), training required a median of 17 epochs (range 13–25) against an epoch budget of 100, so every run stopped on the error threshold rather than on the budget, and the accumulated design matrix reached was on the order of 1.5–3 million rows by the final epoch. On completion, a final deterministic pass converts the diplotype call recorded for each individual into relative haplotype frequency estimates for the reference database used at inference time.

This training strategy departs from the ELM’s typical use case, in which the correct label for each training example is known in advance, and instead resembles the iterative refinement approach used in expectation maximization (EM)-based haplotype-frequency estimators such as HaploStats [10] and Hapl-o-Mat [12], which start from an analogous uniform (or frequency-weighted) prior over compatible diplotypes and iteratively update haplotype frequency estimates [7]. Those EM-based methods have established convergence properties under standard assumptions (e.g., Hardy–Weinberg equilibrium, correct specification of the compatible-diplotype set). We have not established an analogous formal convergence proof for the present random-sampling/closed-form-update training procedure or evaluated alternative initialization strategies (e.g., frequency-weighted rather than uniform priors over compatible diplotypes) against the uniform-probability assumption used here. We consider this a substantive open methodological question rather than a settled property of the method, and we explicitly flag it as a limitation (Section 5): the empirical accuracy reported in Section 3.1 is evidence that the trained network recovers usable diplotype probabilities on this dataset, but it does not by itself establish that the procedure converges to the true underlying haplotype distribution in general.

Algorithms S1 and S2 (Supplementary Section S2) summarize the training and inference procedures described above in pseudocode.

### 2.6. Benchmark Comparators

Framework-level benchmarking of the base ELM and three established extensions (Kernel ELM [25], Weighted ELM [26], and Ensemble ELM [27]) against haplotype frequency imputation, a multilayer perceptron (MLP) [17], and gradient-boosted trees [28,29,30] was performed on a simulated reference dataset derived from the published NMDP US registry haplotype frequency resource [8], unrelated to the HTO/ORAM/GRPT cohorts; the dataset, the extensions, these comparators, and the corresponding results are reported in Supplementary Section S3 (Tables S1–S5, Figures S16 and S17).

On the Greek registry data, we implemented and ran a narrower, two-locus (HLA-A~HLA-B) EM haplotype frequency baseline directly on the HTO/ORAM/GRPT data (Supplementary Section S4.1) and separately obtained and scored full five-locus GRIMM output on the same verified donor-independent ORAM and GRPT genotypes (Section 3.2), a direct, same-dataset, five-locus comparison against an established EM-based tool. An open source HaploStats-inspired baseline (Supplementary Section S4.3) and another in-house five-locus EM baseline approximating Hapl-o-Mat’s core algorithm (Supplementary Section S4.4) were tested on GRPT, ORAM, and the HTO training registry; the published Hapl-o-Mat tool and NMDP HaploStats service itself have still not been run on this data, and this remains an outstanding step for corroborating that GRIMM’s results generalize across EM-based tools rather than being specific to GRIMM’s reference panel and matching algorithm (Section 5).

### 2.7. Validation Strategy, Hyperparameters, and Computational Environment

Model performance on the simulated reference dataset (Supplementary Sections S3.4–S3.6) was assessed using five-fold cross-validation [31,32] stratified by a broad ancestry category, so that each fold preserved the ancestry composition of the full cohort. For every locus, we report top-1 allele-pair concordance (the proportion of individuals for whom the predicted high-resolution genotype exactly matched the confirmatory typing), top-3 concordance (correct genotype present among the three highest-probability predictions), and per-population concordance stratified by the broad ancestry category. For the Greek registry (Section 3.1), five-fold cross-validation on the 117,345-donor HTO dataset was used exclusively to select the hidden-layer size and other hyperparameters and to report internal per-experiment accuracy (Section 3.1, Table 6 and Table 7); it was not applied to, and does not overlap with, the ORAM and GRPT cohorts, which were held out entirely for external validation and were never used for training or hyperparameter selection. For the ELM variant comparison (Supplementary Section S3.7), we additionally report accuracy restricted to alleles below the locus median frequency (‘rare-allele accuracy’) and the standard deviation of top-1 concordance across repeated random initializations. Accuracy and call rate figures throughout this article (Table 7, Table 8, Table 9, Table 10 and Table 11 and Supplementary Tables S2, S4 and S6–S8) are reported with 95% confidence intervals in square brackets alongside each point estimate, given the large cohort sizes available and the need to determine whether differences between loci, experiments, ELM variants, and comparator methods are distinguishable from sampling noise rather than noise alone. Because several of this article’s small-sample comparator results (Supplementary Sections S4.3 and S4.4) sit at or near the 0% or 100% boundary, a direct nonparametric bootstrap [33] is uninformative there (a fully homogeneous sample of size n resamples only itself, giving a degenerate zero-width interval regardless of n); therefore, we computed each interval using the Wilson score method [34], which is asymptotically equivalent to a percentile bootstrap for large, well-behaved samples but remains well-calibrated at small n and at proportions near 0% or 100%, unlike the normal approximation (Wald) interval typically associated with naive bootstrap resampling. Where only a percentage and a sample size were available rather than an exact count, the underlying count was reconstructed by rounding; this applies specifically to Supplementary Tables S2 and S4, the simulated-dataset benchmark of Supplementary Section S3, where per-cell donor counts were not tracked separately. By contrast, Table 7 (the real-registry Experiment 1–5 breakdown) is exact: its per-experiment, per-locus donor counts were extracted directly from the evaluation log. Table 7, Table 8 and Table 9, Supplementary Table S6, and the great majority of cells in Table 10 and Table 11 and Supplementary Tables S7 and S8 report exact numerator/denominator counts directly alongside each percentage, and their intervals were computed from those exact counts rather than by rounding. Where a table reports a range across loci rather than one value, the interval is given for the reported low and high endpoints individually rather than for the range as a whole.

**Figure 2:**
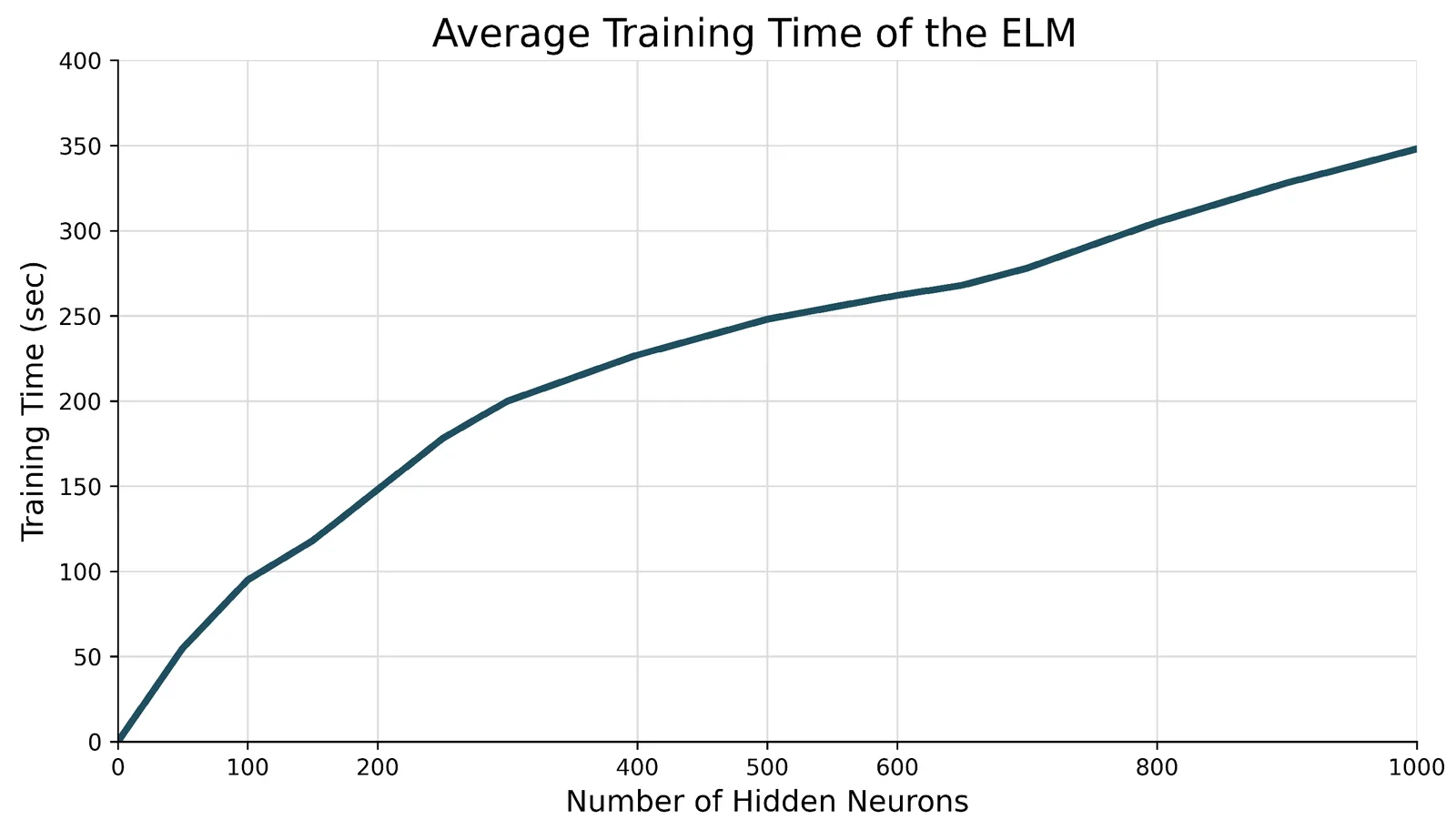
Average ELM training time as a function of hidden-layer size (Greek registry data).

**Figure 3:**
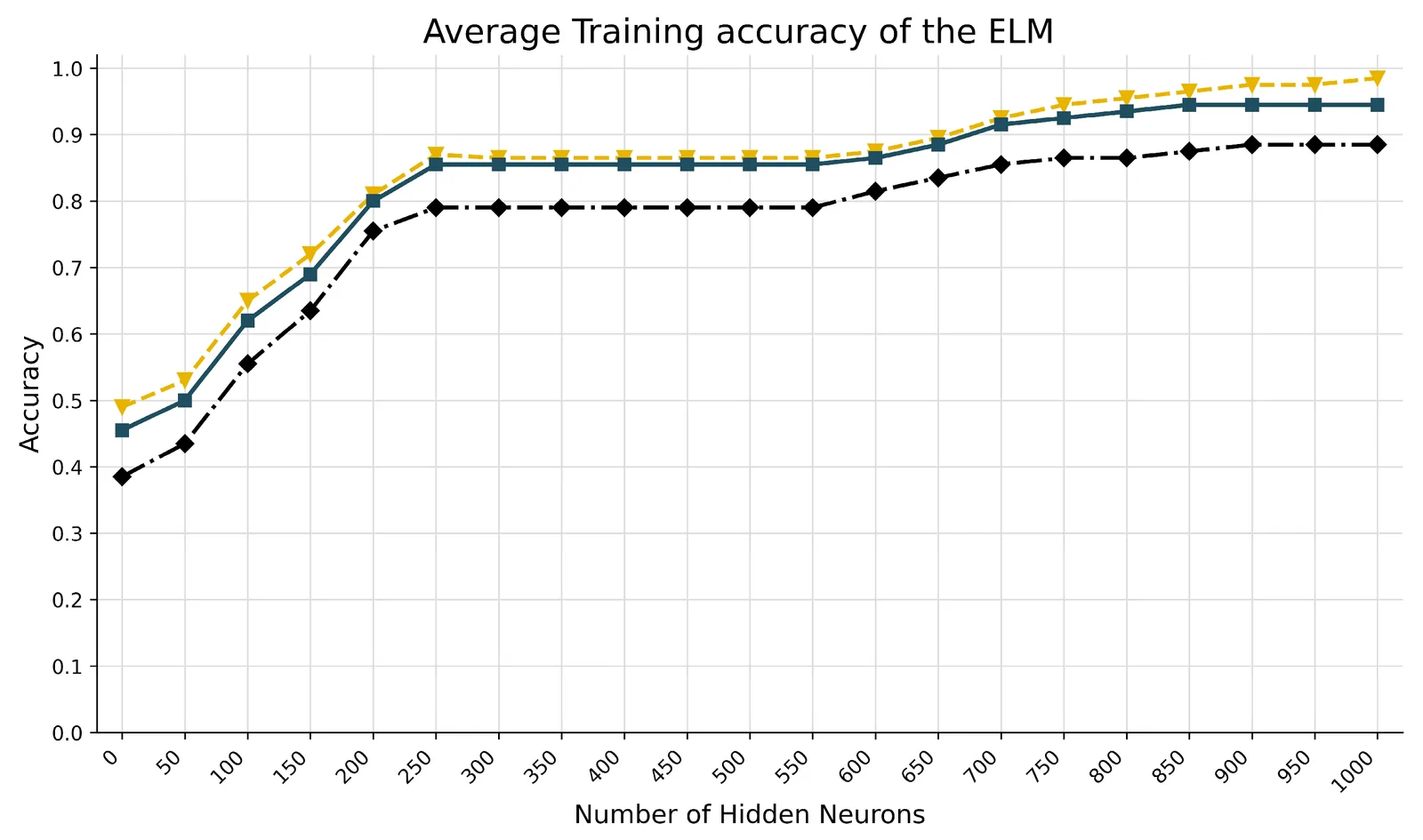
Average ELM training accuracy as a function of hidden-layer size (Greek registry data). The three curves correspond to the three locus combinations evaluated; the plotting symbols distinguish the curves and carry no further meaning.

**Figure 4:**
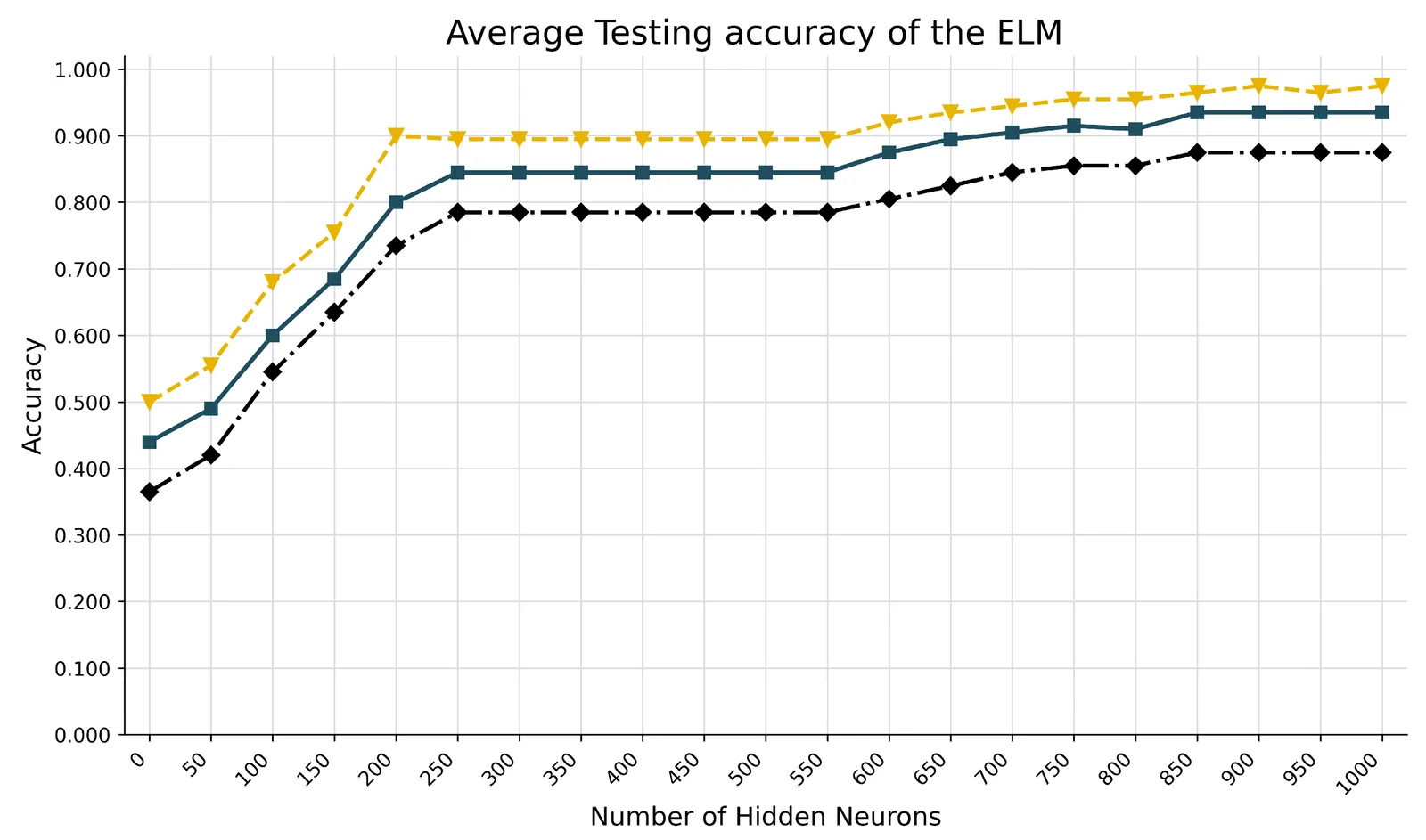
Average ELM testing accuracy as a function of hidden-layer size (Greek registry data); accuracy stabilizes beyond approximately 700 hidden neurons without a subsequent decline, though this alone does not establish absence of overfitting (see text).

The size of the final hidden layer was selected as 750 neurons, the smallest size within the plateau of stable testing accuracy identified in the hidden-neuron sweep (2 to 1000 neurons, 75 repeated runs per size; Section 3.1, Figure 2, Figure 3 and Figure 4), using a sigmoid activation function; hidden-layer weights and biases were redrawn independently for each repeated run rather than from a single fixed random seed so that the reported testing–accuracy curves (Figure 2, Figure 3 and Figure 4) reflect run-to-run variability rather than a single arbitrary initialization. The implementation combined Python (model training and orchestration) with a MySQL backend (haplotype database queries and diplotype enumeration). The CPU, RAM, core count, and operating system are specified below for the reported runtime benchmarks (Section 3.1): all ELM training and inference runs and all benchmark comparator runs (GRIMM, the hlaR ImputeHaplo baseline, the two-locus EM baseline, and the in-house five-locus EM baseline, Supplementary Section S4.4) were executed on the “Titanas” server of the Biomedical Engineering Laboratory, School of Electrical and Computer Engineering, National Technical University of Athens (NTUA). Titanas is configured with a single AMD EPYC 9224 CPU (24 cores, 48 threads; Advanced Micro Devices, Santa Clara, CA, USA), 256 GB of ECC DDR5 RAM, two NVIDIA RTX 6000 Ada Generation GPUs (NVIDIA Corporation, Santa Clara, CA, USA; 48 GB VRAM each), two 3.84 TB Micron 7450 NVMe SSDs, a PCIe 5.0-capable motherboard, and 10 GbE networking; all neural network models reported here were run on only one of the two GPUs, so the runtimes reported in Section 3.1 reflect one-GPU execution. Titanas runs Ubuntu Server 24.04.3 LTS (kernel 6.8.0) with GCC/G++ 13.3.0. All Python code was executed in a dedicated conda environment running Python 3.12, with NumPy 2.5.3, SciPy 1.18.1 and (for the MLP and gradient-boosted-tree comparators of Supplementary Section S3) scikit-learn at the versions recorded in the released code repository; the haplotype database backend was MySQL 8.0.45. Source code for the implementations presented in this article, developed in the context of this work and the associated doctoral thesis, is open source (MIT License) and available from the repository of the Biomedical Engineering Laboratory, National Technical University of Athens, archived on Zenodo (DOI: 10.5281/zenodo.22061923), so that other registries can verify, reproduce, and extend this work, consistent with reproducibility expectations for methodological studies of this kind.

## 3. Results

### 3.1. Validation on the Greek National Registry of Donor Candidates

Definition of an evaluable set for this section: donors with unambiguous two-field (high-resolution) confirmatory ground truth, scored per locus unless stated otherwise.

To assess the framework on a real, single-country registry rather than simulated benchmarking data, a population-specific ELM was trained and validated on the Greek national donor registry maintained by the Hellenic Transplant Organization (HTO). The registry, established in 2002 and populated through recruitment at five donor centers between 2002 and 2019, comprised 117,345 donors, of whom approximately 20% held only low-resolution records. Validation was additionally performed on two independent Greek cohorts external to the training registry: ORAM (*n* = 20,100) and GRPT (*n* = 4353).

Rather than a single fixed prediction task, performance was evaluated across five experiments that progressively increased in difficulty; an increasing number of loci were treated as available only at low-/mid-resolution and required upgrading, while the remaining loci were retained at their known high-resolution values to serve as haplotypic context for the model (Table 6). In Experiment 1, only HLA-A required upgrading, with HLA-B, -C, -DRB1, and -DQB1 already known at high resolution; in Experiment 5, all five loci required simultaneous upgrading with no high-resolution anchor locus available. We state this direction explicitly because the underlying experiment table is easy to misread: the ‘upgraded’ column lists the locus/loci that were evaluated as model output, while the ‘context’ column lists loci whose high-resolution genotype was already known and supplied as input, not loci being predicted. This set of experiments was conducted to evaluate resolution upgrading of loci that were typed (at low-/mid-resolution) in every case; it is distinct from the missing-locus imputation task described in Section 2.2 and Section 2.4, where a locus has no typing at all, which we did not separately tabulate experimentally here.

**Table 6:** Experimental design used for the Greek registry validation: number and identity of loci upgraded from low-/mid- to high-resolution versus loci retained as known high-resolution context, per experiment.

Experiment	Locus/Loci Upgraded (Low-/Mid-Resolution Input)	Locus/Loci Retained as High-Resolution Context
1	HLA-A	HLA-B, -C, -DRB1, -DQB1
2	HLA-A, -B	HLA-C, -DRB1, -DQB1
3	HLA-A, -B, -C	HLA-DRB1, -DQB1
4	HLA-A, -B, -C, -DRB1	HLA-DQB1
5	HLA-A, -B, -C, -DRB1, -DQB1	None

Model performance was assessed via five-fold cross-validation on the full registry (test folds of approximately 23,469 donors against training folds of approximately 93,876 donors), reporting the proportion of donors for whom the upgraded high-resolution genotype exactly matched the confirmatory record at each upgraded locus (Table 7). Successful prediction rates were highest in Experiment 1, where a single locus was upgraded using four known high-resolution loci as context (94% for HLA-A), and declined progressively as more loci required simultaneous upgrading and fewer high-resolution anchors remained, reaching 19–22% per locus in Experiment 5, where all five loci were upgraded from low-/mid-resolution input alone.

**Table 7:** Per-locus successful prediction (upgrade) rate by experiment, with five-fold cross-validation on the HTO registry (117,345 donors). Rates decline as fewer loci remain available for the high-resolution context. The per-experiment, per-locus evaluable-donor counts range from 86,002 to 96,726, consistent with Table 1’s proportions of typing completeness. The 95% confidence intervals shown were computed from exact per-cell k and n extracted directly from the evaluation log.

Experiment	HLA-A	HLA-B	HLA-C	HLA-DRB1	HLA-DQB1
1	94.05% [93.89–94.21%]	–	–	–	–
2	85.01% [84.77–85.24%]	87.02% [86.79–87.24%]	–	–	–
3	72.04% [71.74–72.33%]	73.01% [72.72–73.30%]	71.01% [70.71–71.31%]	–	–
4	61.03% [60.71–61.35%]	61.00% [60.68–61.32%]	62.00% [61.68–62.32%]	65.04% [64.73–65.35%]	–
5	19.01% [18.76–19.27%]	22.02% [21.75–22.29%]	19.01% [18.75–19.27%]	22.02% [21.76–22.28%]	20.01% [19.75–20.27%]

The resolution of the input typing also affected accuracy independently of how many loci required upgrading. This analysis used a different evaluation condition from Experiment 5 above: rather than the low-/mid-resolution input alone evaluated in Table 7 (19–22% per locus, no high-resolution anchor at any locus), every donor’s input was restricted to records with a first-field call present at all five loci simultaneously (a narrower, higher-information subset than Experiment 5 in Table 7, though still below full second-field resolution), with the second-field allele pair as the prediction target. Per-locus accuracy on the HTO training registry (five-fold cross-validation) under this first-field-input condition was 91% (HLA-A), 93% (HLA-B), 94% (HLA-C), 82% (HLA-DRB1), and 89% (HLA-DQB1). The accuracy of the corresponding joint (with all five loci being simultaneously correct) was 59.6% under the same HTO condition, which is reasonably consistent with the 58.1% implied by naively multiplying the five per-locus figures together (a mild positive correlation between loci, consistent with linkage disequilibrium, would be expected to push the true joint accuracy slightly above that naive product). On the external ORAM cohort, per-locus accuracy under the same first-field-input condition was lower for most loci (82%, 91%, 83%, 72%, and 89%, respectively), as expected for a held-out cohort not used in training; the joint accuracy on ORAM is 44.0%, reasonably close to the 39.7% implied by naively multiplying ORAM’s own per-locus figures together (a gap of 4.3 percentage points, which is of the same order as the 1.5-percentage-point gap on the HTO condition above and similarly attributable to a mild positive correlation between loci), verified directly against the underlying per-donor scoring log. Separately, restricting the same first-field-input condition to intermediate- or second-field (high-resolution) input instead increased the joint accuracy to 91.6% and 97%, respectively, consistent with the general pattern that more informative input resolution yields more accurate upgrading.

The accuracy and call rate were also assessed jointly as a function of the posterior probability confidence threshold used to accept a call (Section 2.3), rather than reported as two independent maxima. Accuracy here is first-field top-1 concordance, pooled over typed loci, among donors carrying at least first-field confirmatory typing—the same evaluable-set definition used for GRIMM in Section 3.2—from the same 25-run per-locus models; the call rate was computed over every donor in each cohort (19,535 ORAM, 4353 GRPT) rather than over the evaluable subset, and it was observed to fall monotonically as accuracy rises. Thus, prediction accuracy ranged from 78% to 91% in ORAM and from 70% to 84% in GRPT, as this threshold varied from 0% to 82% posterior probability; over the same range, the call rate fell steadily, with a more pronounced drop once the threshold exceeded approximately 38%, reaching approximately 41% at the 82% threshold. The headline pair reported for the framework as a whole—an overall call rate of 98.1% and an overall two-field accuracy of 50–78%, with a directly computed first-field companion figure of 78–93% (Section 3.2)—comes from a separate, controlled cross-cohort evaluation: five per-locus ELM models, trained on the HTO registry, evaluated on GRPT, ORAM, and HTO itself, and averaged over 25 repeated runs to control for run-to-run variability. This is distinct from the threshold curve and Table 7 figures discussed above, which remain narrower, separately valid experiments in their own right and should not be conflated with this headline pair; none of these figures should be read as simultaneously achievable at one operating point. The appropriate operating point depends on whether a registry prioritizes maximizing the number of donors that receive an upgraded call (low threshold, lower accuracy) or the reliability of the calls it does return (high threshold, fewer donors covered).

Accuracy also varied by allele frequency within a locus. For HLA-C in Experiment 4, common diplotypes such as C*07:01-C*07:01 and C*02:02-C*07:01 were upgraded correctly in over 90% of donors, whereas less frequent combinations such as C*01:02-C*04:01 and C*01:02-C*07:01 were correctly upgraded in approximately two-thirds of donors (Table 8). A comparable frequency-dependent pattern was observed for HLA-B2 (the second of the donor’s two recorded HLA-B alleles, a convenience grouping by recording order rather than a biologically distinguishable second chromosome; Table 9), where the most common Greek allele, B*51:01, was correctly upgraded in 76% of donors, while several less common alleles fell in the 42–66% range.

**Table 8:** Successful upgrade rate for the eight most frequent HLA-C1/C2 diplotypes (C1 and C2 denote the two HLA-C alleles composing each donor’s diplotype), Experiment 4, Greek registry (abbreviated from the source dataset’s 16 most frequent diplotypes).

HLA-C1, -C2	Donors (n)	Correctly Upgraded (n)	Accuracy
04:01–07:01	7254	5875	81% [80.1–81.9%]
04:01–12:03	5300	4240	80% [78.9–81.1%]
07:01–12:03	4819	4047	84% [82.9–85.0%]
04:01–04:01	3934	3422	87% [85.9–88.0%]
07:01–07:01	3419	3145	92% [91.0–92.8%]
02:02–07:01	2876	2617	91% [89.9–92.0%]
01:02–04:01	1694	1134	67% [64.7–69.1%]
01:02–07:01	1623	1119	69% [66.7–71.2%]

**Table 9:** Successful upgrade rate for a subset of the most frequent HLA-B2 alleles (B2 denotes the second of the donor’s two HLA-B alleles), Experiment 4, Greek registry (abbreviated from the source dataset’s 15 most frequent alleles). Because the order in which a donor’s two HLA-B alleles are recorded in a registry file is arbitrary rather than biologically meaningful, the B2 stratification should be read as a convenience grouping by allele identity, not as a property of a distinguishable second chromosome.

HLA-B2	Donors (n)	Correctly Upgraded (n)	Accuracy
51:01	28,054	21,321	76% [75.5–76.5%]
35:01	7606	4792	63% [61.9–64.1%]
44:02	7126	4703	66% [64.9–67.1%]
55:01	7028	4498	64% [62.9–65.1%]
52:01	6404	4163	65% [63.8–66.2%]
18:01	6294	4154	66% [64.8–67.2%]
40:02	3141	1476	47% [45.3–48.7%]
35:02	3094	1299	42% [40.3–43.7%]

A grid search over the size of the hidden layer (2 to 1000 hidden neurons, 75 repeated experiments per size) showed the expected ELM behavior: training accuracy increased monotonically with hidden-layer size, and testing accuracy rose steadily and stabilized once the hidden layer exceeded approximately 700 neurons, without a subsequent decline (Figure 2, Figure 3 and Figure 4). We describe this cautiously rather than as definitive proof of overfitting resistance: a plateau in testing accuracy while training accuracy remains high is consistent with, but does not on its own establish, absence of overfitting, particularly since the allele vocabulary was abridged prior to training (Section 2.4), which shrinks the effective output space and could itself flatten the testing curve independently of any genuine regularization property. ELM’s single-layer, closed-form structure is less prone to overfitting than gradient-trained networks in general, and the large training set relative to the hidden-layer sizes tested (up to 1000 neurons against 117,345 donors) makes substantial overfitting less likely here; nonetheless, a more rigorous generalizability check (e.g., nested cross-validation, or testing accuracy behavior with the full, non-abridged allele vocabulary) would be needed to support a stronger claim. Training a 1000-neuron model on the full registry required approximately 350 s (Figure 2, Figure 3 and Figure 4).

**Figure 5:**
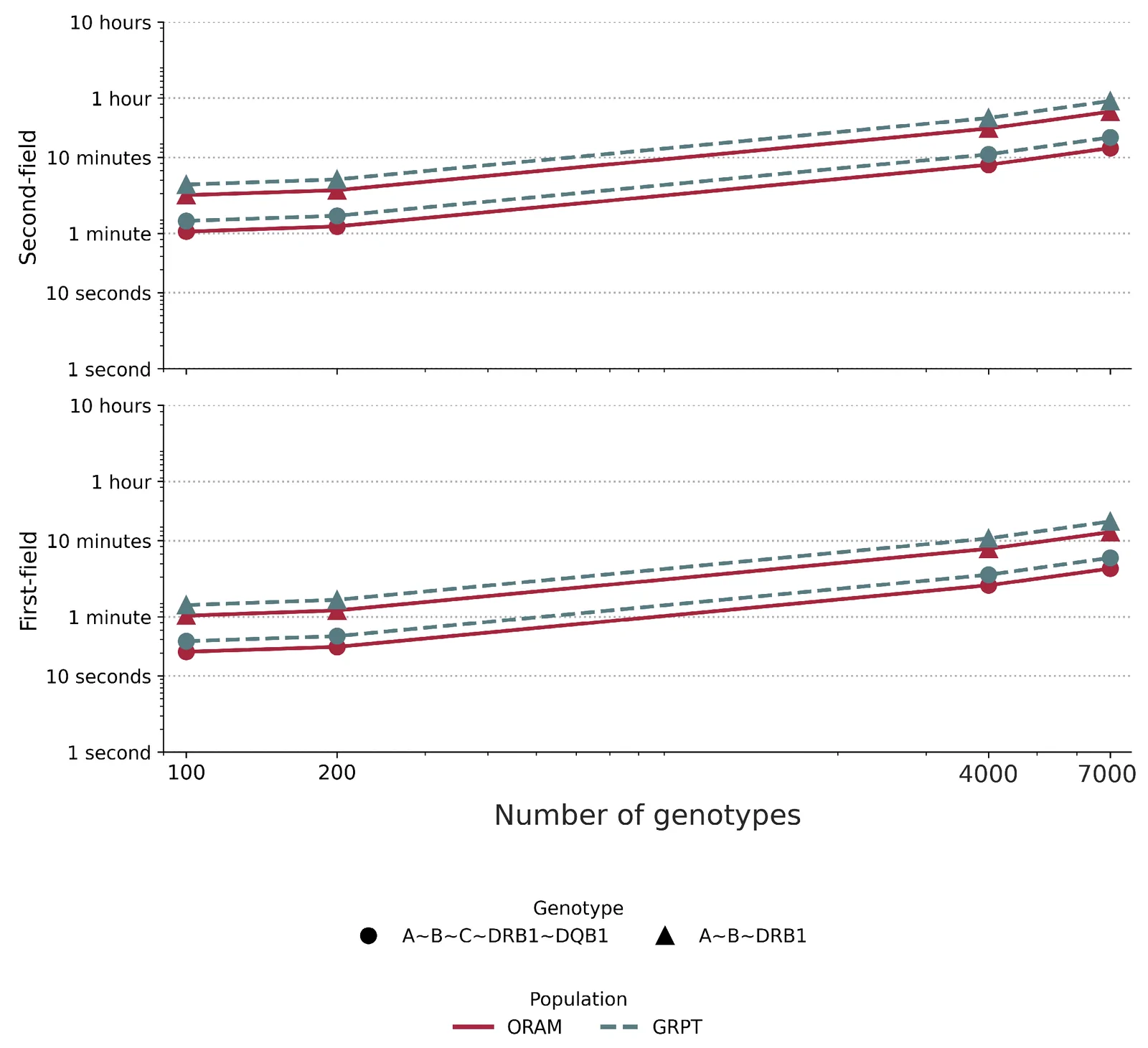
ELM execution runtime by input file size, resolution, and locus combination, evaluated on the ORAM and GRPT cohorts.

Runtime scaled approximately linearly with input file size across both the ORAM and GRPT cohorts, tested at 100, 200, 4000, and 7000 genotypes, two resolutions (first- and second-field), and two locus combinations (HLA-A~B~DRB1 and HLA-A~B~C~DRB1~DQB1). A 100-genotype first-field HLA-A~B~DRB1 batch of Greek ancestry donors was processed in 73 s, rising to 15.2 min for larger first-field batches and 46.7 min for second-field batches of comparable size, reflecting the larger number of candidate haplotypes that must be evaluated at second-field resolution. Runtime was also sensitive to the size of the reference population database and to the number of loci considered jointly; in this dataset, the three-locus A~B~DRB1 combination required roughly three times longer to run than the five-locus A~B~C~DRB1~DQB1 combination (Figure 5), which is a counterintuitive result given that the theoretical candidate diplotype space grows, rather than shrinks, with each additional locus. A plausible explanation, which we flag as a hypothesis rather than a confirmed mechanism, is that the reference-database lookup benefits from additional loci as query constraints and is not burdened by them: with all five loci specified, the database query is highly selective and can narrow the candidate haplotype set immediately, whereas with only three loci specified, the remaining two loci stay unconstrained and a substantially larger set of partially matching haplotypes must be evaluated before a call can be returned. This would be consistent with standard constrained-query database behavior (more constraints narrow the search space faster than the nominal combinatorial growth in candidates); direct profiling of the query execution, rather than the aggregate wall-clock timing reported here, is needed to confirm this specific mechanism and is identified as a follow-up benchmarking task (Section 5).

### 3.2. Head-to-Head Comparison Against GRIMM, Run Directly on ORAM and GRPT

Definition of the evaluable set for this section: GRIMM’s own output is scored at first-field resolution against confirmatory ground truth at whatever resolution is available per donor per locus, which is a broader (first-field, not necessarily two-field) evaluable set than the one used in Section 3.1 and Supplementary Section S4.1; joint accuracy is computed only over the subset of loci for which a given donor has both a GRIMM call and ground truth, which need not be the same five loci for every donor.

GRIMM [11] five-locus (HLA-A, -B, -C, -DQB1, -DRB1) imputation output was obtained for the same verified donor-independent ORAM and GRPT genotypes used throughout this article, providing a direct, same-dataset, five-locus comparison against an established EM-based tool. For each donor, GRIMM reports a status (“imputed” or “no_match_in_reference_panel”) and, when imputed, a top-1 five-locus diplotype with an associated (unnormalized) likelihood. We matched GRIMM’s output back to the confirmatory typing in ORAM/GRPT by donor identifier (full match for GRPT; for ORAM, the raw file’s full donor identifier (GRID) was used to exclude both duplicate records and records with a missing identifier prior to matching: Of the 20,100 raw ORAM records, 300 were duplicate GRIDs (resolved by keeping the first occurrence) and 265 had no GRID recorded (excluded, since they cannot be reliably matched), leaving 19,535 uniquely identified ORAM donors, which is the denominator used for the ORAM call rates in Section 3.2 and Section 3.3 and Supplementary Sections S4.2–S4.4. The two-locus baseline of Supplementary Section S4.1 is scored on a narrower evaluable set and states its own denominator and scored top-1 concordance at first-field (broad antigen-group) resolution, since GRIMM’s output mixes first- and second-field calls across loci and donors and first-field is the common denominator across all of them. This makes the comparison below asymmetric in one specific, identifiable direction: GRIMM (and, in Supplementary Sections S4.3 and S4.4, hlaR ImputeHaplo and the in-house EM baseline) is scored at first-field resolution throughout, while the ELM framework’s own headline accuracy range (50–78%; Section 3.1) is a two-field, exact-match figure. Because a correct two-field call is by definition also a correct first-field call (the reverse does not hold), the ELM’s true first-field accuracy can only be equal to or higher than its two-field figure at every operating point. Rather than rely on that logical bound alone, we directly computed the ELM’s first-field accuracy: five per-locus models, using the domain-structured topology described in Section 2.4, were trained on the 117,345-donor HTO registry (per-locus training sets of 87,830–96,917 donors with high-resolution ground truth) and evaluated on the verified donor-independent ORAM and GRPT cohorts, with results averaged over 25 repeated runs (independently redrawn, random hidden-layer weights per run, as in the hidden-neuron sweep of Section 3.1/Figure 4) to control for run-to-run variability. The resulting first-field accuracy was 78–91% on ORAM and 85–93% on GRPT across the four loci with more than a handful of evaluable donors (100% at HLA-DQB1, *n* = 4), and 80–94% on the HTO training registry itself; the per-locus figures, with their exact numerators and denominators, are given in Supplementary Table S9. The comparator accuracy figures below are compared directly against these first-field figures rather than against the two-field range.

GRIMM’s call rate was far lower than the ELM framework’s reported figure on both cohorts: 47.9% on ORAM (9358/19,535) and only 1.8% on GRPT (78/4353), against the ELM’s reported overall call rate of 98.1% (Table 10, Figure S5). The low GRPT call rate is consistent with the finding reported in the caption of Table 4 (Section 2.4), stating that 99.8% of GRPT donors are typed only at ambiguous, low-resolution input at HLA-A: GRIMM’s reference-panel matching evidently requires more typing information than most GRPT records provide, but the ELM framework is specifically designed to accept exactly this kind of sparse input. Among the donors GRIMM did call, however, first-field top-1 accuracy was similar to, and on several loci above, the ELM’s own first-field accuracy (78–91% on ORAM, 85–93% on GRPT): GRIMM achieved 80.3–99.9% per locus on ORAM (*n* = 9342 evaluable donors, a subset of the 9358 called donors that also have confirmatory ground truth) and 97.2–100% on GRPT (*n* = 77, a small sample that should be treated cautiously), with a joint (all evaluable loci at the same time) accuracy of 74.9% on ORAM and 97.4% on GRPT (Table 10, Figure S6). We also checked whether GRIMM’s reported likelihood predicts accuracy the way the ELM’s normalized posterior probability does: when splitting ORAM’s imputed donors into quartiles of log-likelihood, the lowest-likelihood quartile had the highest joint accuracy (92.0%) and the two middle quartiles had the lowest (67.9–75.1%), which suggests a non-monotonic relationship. This indicates that GRIMM’s raw likelihood, as an unnormalized product of reference haplotype frequencies rather than a posterior probability over the compatible set, is not directly usable as a calibrated confidence threshold in the way the ELM’s Post-P is, and any future joint accuracy/call rate curve comparison between the two methods should account for this difference in what the reported probability represents rather than treat the two scales as equivalent.

**Table 10:** GRIMM five-locus imputation performance on the same verified donor-independent ORAM and GRPT cohorts used throughout this study, first-field top-1 concordance. Joint accuracy is scored per donor over whichever loci that donor has both a GRIMM call and ground truth for, which need not be the same five loci across donors (Section 3.2); it is not a fixed five-locus requirement despite the shorthand name of the column. GRPT figures rest on a small evaluable sample (*n* = 77) because GRIMM’s reference-panel matching succeeded for very few GRPT donors, which is consistent with GRPT being typed mostly at an ambiguous, low resolution (Table 4, Section 2.4).

Cohort	Call Rate	Per-Locus Accuracy Range	Joint Accuracy (Per-Donor Evaluable Loci)	n Evaluable
ORAM	47.9% (9358/19,535) [47.2–48.6%]	80.3–99.9% [endpoints’ 95% CI: 79.5–81.1% to 99.8–99.9%]	74.9% [74.0–75.8%]	9342
GRPT	1.8% (78/4353) [1.4–2.2%]	97.2–100% [per-locus n varies; endpoint intervals not reported]	97.4% [91.0–99.3%]	77

**Figure 6:**
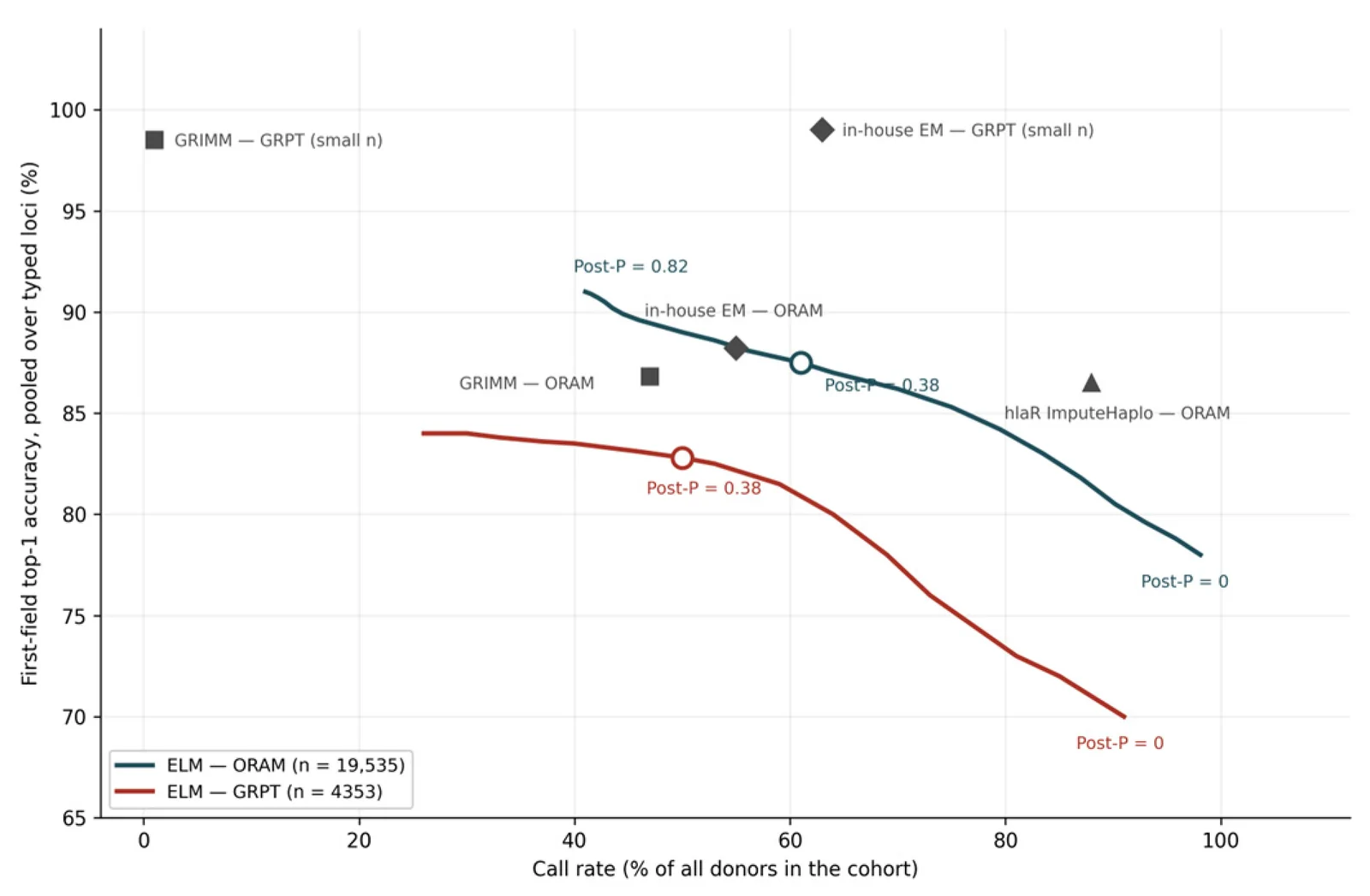
Accuracy/call rate trade-off on the external validation cohorts. Curves trace the ELM framework as the posterior probability threshold rises from 0 to 0.82 (open circles mark the 0.38 knee); accuracy is first-field top-1 concordance pooled over typed loci among donors with confirmatory typing, and call rate is computed over all donors in each cohort. Gray markers denote the haplotype frequency comparators scored identically on the same cohorts: GRIMM (Section 3.2), hlaR ImputeHaplo and the in-house five-locus EM baseline (Section 3.3, Supplementary Sections S4.3 and S4.4). GRPT comparator points rest on very small evaluable samples (*n* = 77 for GRIMM, *n* = 2–45 for the in-house EM baseline) and are illustrative only.

Figure 6 places both approaches on the same axes. Taken together, this head-to-head comparison shows that the two approaches are broadly comparable in a specific, quantified sense: on this registry, GRIMM and the ELM framework trade off in opposite directions along the accuracy/call rate curve, with GRIMM achieving a similar or somewhat higher top-1 accuracy on the minority of donors it is willing to call, and the ELM framework achieving a far higher call rate, particularly on sparser, lower-resolution input such as GRPT. Runtime was not reported in the GRIMM output file and so it is not compared here. GRIMM is not distributed as a versioned software package; the imputation output analysed here was obtained as a data file, and no version identifier was recorded in it. GRIMM was run with its default public NMDP US haplotype frequency reference panel (European/Caucasian population) and default parameters, using the same configuration for the HTO, ORAM, and GRPT runs. That panel is US-derived and therefore not population-matched to the Greek cohorts, a point further discussed in Section 5. Notably, hlaR ImputeHaplo, which draws on a reference table from the same US resource, called 88.7% of ORAM donors (Supplementary Section S4.3), so GRIMM’s much lower call rate reflects its stricter handling of ambiguous or incomplete input rather than the reference population alone. Two other haplotype frequency baselines are reported in Section 3.3 and Supplementary Sections S4.3 and S4.4; Hapl-o-Mat and the NMDP HaploStats service itself have not been run on these data (Section 5).

### 3.3. Comparison with Other Haplotype Frequency Baselines

Three other haplotype frequency baselines were run on the same cohorts (Section 2.6): a two-locus (HLA-A~HLA-B) EM baseline, an open-source HaploStats-inspired baseline (hlaR ImputeHaplo [35]), and an in-house Hapl-o-Mat-style five-locus EM baseline. Their full descriptions and per-cohort results are reported in Supplementary Sections S4.1, S4.3 and S4.4 (Tables S6 and S8, Figures S3, S4 and S8–S15), and GRIMM was applied to the HTO training population itself, as discussed in Supplementary Section S4.2 (Table S7, Figure S7). On ORAM, the two-locus EM baseline returned a call for 53.9% of donors (1243/2305), with 4.7% top-1 accuracy among called donors (58/1243; Table S6). Table 11 summarizes the ELM, GRIMM, hlaR ImputeHaplo, and in-house five-locus EM baseline across the three cohorts.

**Table 11:** Method comparison across GRPT, ORAM, and HTO: ELM (reported) vs. GRIMM (Section 3.2, Supplementary Section S4.2) vs. hlaR ImputeHaplo (Supplementary Section S4.3) vs. this in-house Hapl-o-Mat-style five-locus EM baseline (Supplementary Section S4.4), first-field top-1 concordance. GRIMM’s own ORAM and HTO results are not duplicated here as separate rows; they are reported in full in Table 10 and Supplementary Table S7 (Section 3.2, Supplementary Section S4.2) and referenced in the discussion below. The ELM’s per-locus accuracy range is reported at first-field resolution (five per-locus models trained on the 117,345-donor HTO registry and evaluated on GRPT/ORAM; results were averaged over 25 repeated runs; Section 3.2). The GRPT comparator results rest on very small evaluable samples for at least some loci (*n* = 2–64) and should be treated as illustrative rather than statistically definitive. The ORAM and HTO comparator results, by contrast, rest on evaluable samples ranging from several hundred to tens of thousands of donors per locus, extracted and verified directly against the underlying per-donor scoring log; they are reported here as statistically robust findings in their own right. The 98.1% call rate is a single overall figure reported once for the ELM framework as a whole (Section 3.1), not measured separately per cohort; it is repeated in each ELM row here only for ease of comparison against the cohort-specific comparator rows. The in-house EM baseline’s evaluable-donor counts on ORAM and HTO are consistent with the high-resolution proportions reported in Table 1. ORAM’s per-locus accuracy is not uniform across loci (26.25–92.45%), comparable in spread to hlaR ImputeHaplo (Supplementary Table S8). HLA-DQB1 has evaluable calls on both ORAM (*n* = 916, k = 361) and HTO (*n* = 42,738, k = 40,542; 94.86%), part of a non-uniform 90.74–97.52% per-locus range on HTO (Supplementary Section S4.4).

Method	Call Rate	Per-Locus Accuracy Range	Joint Accuracy	n Evaluable (Joint)	Loci Tested
ELM (reported)—GRPT	98.1%	85–93%	n/a	n/a	All 5
ELM (reported)—ORAM	98.1%	78–91%	n/a	n/a	All 5
ELM (reported)—HTO	98.1%	80–94%	n/a	n/a	All 5
GRIMM—GRPT (Section 3.2)	1.8% (78/4353) [1.4–2.2%]	97.2–100% [per-locus n varies; endpoint intervals not reported]	97.4% [91.0–99.3%]	77	All 5
hlaR ImputeHaplo—GRPT (Section S4.3)	98.3% (4278/4353) [97.8–98.6%]	18.2–90.6% [endpoints’ 95% CI: 5.1–47.7% to 81.0–95.6%]	0.0% [0.0–65.8%]	2	All 5 (*n* = 2–64 per locus)
In-house 5-locus EM baseline—GRPT (Section S4.4)	63.8% (2777/4353) [62.4–65.2%]	100.0% (*n* = 2–45) [*n* = 2: 34.2–100.0%; *n* = 45: 92.1–100.0%]	100.0% [92.1–100.0%]	45	All 5 (DQB1: no evaluable call)
In-house 5-locus EM baseline—ORAM (Section S4.4)	55.5% (10,847/19,535) [54.8–56.2%]	26.25–92.45% [endpoints’ 95% CI: 24.40–28.19% to 91.64–93.20%]	6.46% [4.88–8.51%]	712	All 5
In-house 5-locus EM baseline—HTO (Section S4.4)	48.00% (56,326/117,345) [47.71–48.29%]	90.74–97.52% (per locus—see Supplementary Section S4.4 for full n/k breakdown)	90.44% (37,101/41,023) [90.15–90.72%]	41,023	All 5

Taken together, Section 3.2 and Supplementary Sections S4.2–S4.4 provide three independent, same-cohort data points on each of GRPT, ORAM, and HTO: GRIMM (lowest call rate on GRPT and HTO, intermediate on ORAM; high accuracy on the small-to-moderate samples it does call), hlaR ImputeHaplo (highest call rate on all three cohorts, scored on evaluable samples ranging from very small on GRPT through one to seven thousand per locus on ORAM to tens of thousands on HTO), and this in-house Hapl-o-Mat-style baseline (intermediate call rate on all three cohorts; a very small, illustrative-only evaluable sample on GRPT versus much larger, verified samples on ORAM and HTO). The relative ordering of call rates (hlaR ImputeHaplo > in-house 5-locus EM baseline > GRIMM) is consistent across all three cohorts. The HTO figures should be read with the caveat that HTO is also the ELM’s training registry and the source population for the in-house EM baseline’s frequency estimates, so neither has been externally validated in this respect; the ORAM and GRPT figures carry the external validation weight. Joint (multilocus) accuracy for the in-house EM baseline differs sharply between HTO (90.44%, per-locus range 90.74–97.52%) and ORAM (6.46%, driven down by markedly lower and more variable per-locus accuracy, 26.25–92.45%); this cross-cohort contrast is a substantive finding in its own right regarding how much registry-to-registry variation this baseline is subject to. For a fully firm ranking of the three comparators, ideally, direct runs of the published Hapl-o-Mat tool and the NMDP HaploStats service itself should be conducted, since neither of our approximations fully resolves that question. We continue to recommend running those specific tools and obtaining validation cohorts with more donors carrying confirmatory two-field ground truth, until a statistically firm ranking of methods on this registry can be established.

## 4. Discussion

The central motivation for this work is practical: registries hold vast numbers of donor records typed at a resolution that no longer matches the requirements of modern matching and mismatch assessment algorithms, and complete re-typing is not feasible. Our results suggest that an ELM-based upgrading pipeline can recover high-resolution genotypes with concordance broadly comparable to established haplotype frequency imputation, while offering a substantial advantage in training speed that makes frequent, near-real-time retraining practical as new confirmatory high-resolution typings accrue.

In our benchmarking, the gradient-boosted tree ensemble achieved the highest concordance at every locus, with the largest margins at HLA-B and HLA-DRB1, the two most polymorphic loci (Supplementary Section S3.4); this is consistent with the machine learning literature showing that tree ensembles handle high-cardinality categorical interactions well. The appeal of the ELM approach lies less in outperforming every alternative on raw accuracy and more in its training time economics: a registry could, in principle, retrain locus-specific ELM models nightly as new high-resolution confirmations arrive, progressively improving imputation quality for rare and previously under-represented haplotypes without the computational burden of recomputing full haplotype frequency tables or retraining an iterative neural network from scratch. Online sequential ELM variants [36] offer an additional route to incremental updating as new confirmatory typings arrive and are a natural extension of the work reported here. One aspect of these training time economics deserves a specific caveat: the three-locus HLA-A~B~DRB1 combination took longer to run than the full five-locus combination in our runtime benchmark (Section 3.1, Figure 5), the opposite of what the larger nominal candidate diplotype space for five loci would suggest. We tentatively attribute this to additional loci acting as query constraints that narrow the reference-database search faster than the combinatorial candidate space grows; the direct profiling of the query execution to confirm this mechanism, instead of inferring it from the aggregate timing alone, is left as a specific follow-up benchmarking task (Section 5).

The disproportionate improvement observed in under-represented ancestry categories is potentially clinically relevant, since these are the populations for whom haplotype frequency imputation is currently least reliable and for whom unrelated donor searches are already the most challenging; we note, however, that this finding comes from the simulated benchmarking dataset (Supplementary Section S3.1), a simulated dataset generated from the published NMDP resource [8] per locus based on the availability of confirmatory high-resolution typing while covering that resource’s six standard ancestry categories, rather than from the Greek HTO/ORAM/GRPT registry data; it has also not been separately confirmed on the latter. If confirmed via prospective validation, this property would make ELM-based upgrading a useful complement, rather than a wholesale replacement, for existing imputation services, for example, as an ensemble input alongside haplotype frequency estimates in the same manner increasingly explored for machine-learning-assisted immunologic risk assessment [37], with disagreement between methods flagged for prioritized confirmatory high-resolution typing.

These general observations are corroborated by validation on the Greek national donor registry (Section 3.1), where a population-specific ELM trained on 117,345 HTO donors and independently validated on the ORAM and GRPT cohorts achieved per-locus accuracy that depended predictably on two factors: how much high-resolution context remained available at the other loci (94% for HLA-A when four other loci were already known at high resolution, falling to 19–22% per locus when all five loci required simultaneous upgrading with no anchor locus), and the posterior probability confidence threshold applied (78–91% accuracy in ORAM and 70–84% in GRPT as the threshold rose from 0% to 82%, with call rate falling accordingly from near-complete coverage to approximately 41%). We report these as a joint accuracy/call rate/context relationship rather than as two independent headline numbers, since accuracy and call rate cannot both be maximized simultaneously at a single operating point (Section 2.3). Taken together, this underscores that the value of ELM-based upgrading is greatest for partially, rather than entirely, low-resolution records, and that any deployment must select and disclose a specific confidence threshold appropriate to its intended use rather than quote best-case accuracy and call rate together.

Consistent with the probabilistic nature of these calls, we emphasize that upgraded or imputed high-resolution genotypes are intended to prioritize and narrow unrelated donor searches, for example, by ranking candidate donors before confirmatory typing is ordered, rather than to substitute for confirmatory high-resolution laboratory typing before a donor is selected for donation or transplantation. Language elsewhere in this article describing the method as ‘increasing the usable donor pool’ should be read in this sense, not as a claim that imputed genotypes are clinically equivalent to laboratory-confirmed ones.

The practical applications that follow from a validated upgrading pipeline of this kind consist of narrowing the pool of candidate donors requiring costly confirmatory high-resolution typing during an active unrelated donor search and providing registries with a rapid, low-cost mechanism with which to assess the population-level impact of resolution upgrades before committing to large-scale re-typing programs.

Three caveats bound how far the comparator results can be pushed. First, the GRPT comparator samples are very small (*n* = 2–64 per locus for hlaR ImputeHaplo, *n* = 2–45 for the in-house EM baseline, *n* = 77 for GRIMM), so the GRPT columns of Table 11 are illustrative only, and no ranking should be read off them; the ORAM and HTO evaluable samples, which are larger by one to four orders of magnitude, carry the comparison. Second, the comparators are scored at first-field resolution, while the framework’s headline accuracy range is a two-field figure, which is why the ELM’s own first-field accuracy was computed directly (Section 3.2) rather than being compared across resolutions. Third, accuracy figures apply to alleles inside the training vocabulary; for donors carrying alleles outside it, the model cannot represent the true call at all (Section 5).

## 5. Limitations

This work has several limitations, several of which we regard as prerequisites for operational use rather than minor caveats.

Incomplete comparator coverage. The framework was benchmarked against GRIMM, an open-source HaploStats-inspired baseline (hlaR ImputeHaplo), and an in-house Hapl-o-Mat-style EM baseline on GRPT, ORAM, and the HTO training registry, but the published Hapl-o-Mat tool and NMDP HaploStats service itself were not run. The comparison against an MLP and gradient-boosted tree ensemble (Supplementary Sections S3.4–S3.6) rests on a simulated reference dataset rather than on the Greek registry data. Of the comparators that were run on registry data, the two-locus (HLA-A~HLA-B) EM haplotype frequency baseline (Supplementary Section S4.1) was substantially outperformed by the ELM framework on the missing-locus imputation task, and the five-locus GRIMM comparison (Section 3.2) is the most informative: GRIMM’s call rate was far below the ELM’s reported 98.1% (47.9% on ORAM, 1.8% on GRPT), while its top-1 accuracy among called donors (80.3–99.9% on ORAM, 97.2–100% on GRPT, small-N caveat) was comparable to the ELM’s own first-field accuracy on the same cohorts, directly computed (78–91% on ORAM, 85–93% on GRPT; Section 3.2). The two approaches are therefore broadly comparable but trade off in opposite directions along the accuracy/call rate curve. An open source, HaploStats-inspired baseline (hlaR ImputeHaplo, Supplementary Section S4.3) and another in-house five-locus EM baseline approximating Hapl-o-Mat’s core algorithm (Supplementary Section S4.4) were tested on GRPT, ORAM, and the HTO training registry: call rates ranged from 53.9 to 98.3% for hlaR ImputeHaplo and from 48 to 63.8% for the in-house baseline across the three cohorts, both consistently higher than GRIMM’s call rate on the corresponding cohort. Per-locus accuracy samples remain small throughout for GRPT (*n* = 2–64 for hlaR ImputeHaplo, *n* = 2–45 for the in-house baseline, with zero evaluable calls at HLA-DQB1 for the latter). On ORAM and HTO, both comparators’ evaluable samples are substantially larger (hlaR ImputeHaplo: *n* = 1285–6930 on ORAM, *n* = 47,132–51,864 on HTO, per locus; in-house EM baseline: *n* = 916–4387 on ORAM per locus with a five-loci joint of *n* = 712, *n* = 41,023 five-loci joint on HTO). This mix of small GRPT samples and much larger ORAM/HTO samples for both comparators is not sufficient to rank the three haplotype-frequency-based comparators with statistical confidence, but the ORAM/HTO results allow for a substantive comparison on their own (Supplementary Sections S4.3 and S4.4), revealing that the in-house baseline’s joint accuracy varies far more across cohorts (6.46% on ORAM vs. 90.44% on HTO) than either comparator’s call rate does. The published Hapl-o-Mat tool and the NMDP HaploStats service itself remain untested on any cohort. Hapl-o-Mat is open source and is used to estimate haplotype frequencies from the user’s own genotypes, so running it directly on the HTO registry is feasible and the appropriate next step; the NMDP HaploStats service exposes no batch interface for a cohort of this size, which is why the open source hlaR ImputeHaplo baseline, which draws on the same NMDP frequency resource, was used in its place (Supplementary Section S4.3). Until these runs are carried out, whether GRIMM’s reference panel and matching algorithm are representative of EM-based tools generally remains unresolved. GRIMM was additionally run with its default US-derived reference panel, which is not population-matched to the Greek cohorts (Section 3.2); whether GRIMM supplied with Greek-derived haplotype frequencies would call a higher proportion of donors has not been evaluated, and the comparison reported here should be read as GRIMM in its default, out-of-the-box configuration. GRIMM’s reported likelihoods were also found to be non-monotonically related to accuracy on these data (Section 3.2) and are not a calibrated confidence measure comparable to the ELM’s Post-P, so a full accuracy/call rate curve comparison (rather than the single operating point compared here) has not yet been performed. A formal head-to-head runtime comparison against GRIMM, hlaR ImputeHaplo, the two-locus EM baseline, and the in-house five-locus EM baseline has not yet been reported; all were run on the same Titanas server as the ELM benchmarks (Section 2.7), which makes such a comparison feasible, and we identify capturing and reporting these comparator runtimes as a specific, well-defined follow-up task.

Quantified allele abridgment bias, greater than the within-registry estimate alone would suggest. Restricting the training vocabulary to the minimum set of alleles needed to describe the training cohort biases the model toward common alleles by construction. Within the HTO registry itself, a vocabulary covering 99% of allele calls excludes 58–81% of the distinct alleles observed per locus (driven overwhelmingly by singleton and near-singleton alleles) while excluding under 1% of allele calls (Section 2.4, Table 5). Applying that same HTO-derived vocabulary to the verified donor-independent ORAM and GRPT cohorts (Table 4) shows that the practical impact is substantially larger than the within-registry figure implies: 18–47% of ORAM allele calls and, excluding HLA-DQB1 (0.0%, near-untyped in GRPT; Section 2.1), 14–22% of the (small-N) unambiguously typed GRPT calls fall outside the training vocabulary, depending on locus. Counted per donor rather than per call, the affected fraction is greater still (26–71% of ORAM donors per locus), and accuracy stratified accordingly (Supplementary Table S9) shows what it costs: 94.2% first-field and 92.8% two-field accuracy for in-vocabulary donors on ORAM, against 73.7% first-field and, by construction, zero two-field accuracy for the rest. The reported headline figures are therefore a weighted average across both strata rather than a description of performance on donors carrying rare alleles. Because rare HLA variants are frequently the most clinically consequential in unrelated donor matching, and because this cross-cohort exclusion rate is far higher than the training registry’s self-consistency figure would suggest, we consider the abridged vocabulary’s generalization to external cohorts a material, only partly mitigated, limitation. Reported accuracy and call rate figures should therefore be read as weighted averages across both strata rather than as performance on either one, and the stratified figures of Supplementary Table S9 are the ones that should guide the search strategy for donors with uncommon alleles.

Training procedure without a formal convergence guarantee. The training labels used for ambiguous training genotypes were sampled under an assumed uniform distribution over compatible diplotypes, refined only implicitly through the iterative sampling and closed-form update procedure described in Section 2.5. Unlike the EM-based methods this framework was compared against, we have not established formal convergence properties for this procedure, or evaluated alternative (e.g., frequency-weighted) initialization strategies. The empirical accuracy reported here demonstrates that the trained network is useful on this dataset, but not that the procedure is guaranteed to recover the true haplotype distribution in general.

Overfitting claim not fully established. The testing accuracy plateau reported in Section 3.1 and Figure 4 is consistent with, but does not by itself establish, resistance to overfitting: a plateau of this kind can also arise because the abridged output vocabulary (Section 2.4) bounds the space of representable predictions independently of any genuine regularization property of the network. Nested cross-validation and a comparison of the same testing accuracy curve computed against the full, non-abridged allele vocabulary, would be needed to disentangle these two explanations, and this is identified here as specific, well-defined follow-up work, as we do not consider it resolved.

## 6. Conclusions

An Extreme Learning Machine framework can upgrade low- and intermediate-resolution donor HLA genotypes to probabilistic high-resolution calls and impute entirely untyped loci at a favorable computational cost relative to the iterative comparators evaluated on a simulated reference dataset (Supplementary Section S3). On a national registry (117,345 Greek donors) and two independent external cohorts, accuracy depended predictably on the amount of high-resolution context available per donor and on the confidence threshold applied, ranging from roughly 19% (five loci simultaneously upgraded, no threshold) to over 90% (a single locus upgraded with several known high-resolution anchors, or a high confidence threshold applied at the cost of the call rate). These calls are intended to prioritize and narrow unrelated donor searches, not to substitute for confirmatory high-resolution typing before donor selection. A direct, same-dataset, five-locus comparison against GRIMM was carried out on the verified donor-independent ORAM and GRPT cohorts: GRIMM achieved a far lower call rate than the ELM’s reported figure (47.9% on ORAM, 1.8% on GRPT, vs. 98.1%) and a competitive-to-higher top-1 accuracy among the donors it called (80.3–99.9% on ORAM, 97.2–100% on GRPT vs. the ELM’s own directly computed first-field accuracy of 78–91% on ORAM and 85–93% on GRPT; Section 3.2), indicating that the two approaches trade off in opposite directions along the accuracy/call rate curve rather than one strictly dominate the other. An open-source HaploStats-inspired baseline (hlaR ImputeHaplo) and an in-house Hapl-o-Mat-style five-locus EM baseline were additionally tested on GRPT, ORAM, and the HTO training registry, both achieving consistently higher call rates than GRIMM on all three cohorts. On GRPT, where evaluable samples are small, accuracy is generally lower than that of GRIMM, while on the much larger ORAM and HTO evaluable samples (Supplementary Sections S4.3 and S4.4), the per-locus accuracy for hlaR ImputeHaplo ranges from 26.30 to 89.53% on HTO and from 28.63 to 93.65% on ORAM, and the joint accuracy for the in-house EM baseline is 90.44% on HTO versus 6.46% on ORAM, a substantial cross-cohort contrast driven by ORAM’s markedly lower and more variable per-locus accuracy (26.25–92.45%). Hapl-o-Mat and the NMDP HaploStats service itself specifically remain untested, so it is not yet known whether this pattern is specific to GRIMM’s reference panel or general to EM-based tools; a narrower two-locus EM baseline, implemented and run directly on this registry’s data, was substantially outperformed by the ELM framework, but these results should not be over-generalized to the full five-locus tools it approximates. Two limitations bound these conclusions. First, cross-cohort allele vocabulary abridgment is materially greater than the within-registry estimate implies (18–47% of ORAM and 14–22% of GRPT allele calls, and 26–71% of ORAM donors per locus, fall outside the training vocabulary), and accuracy stratified on that basis falls from 94.2% to 73.7% at first field on ORAM, with a two-field accuracy of zero outside the vocabulary (Supplementary Table S9); the training procedure, unlike the EM-based methods it is compared against, has no formal convergence guarantee. Second, establishing convergence behavior empirically remains necessary before the framework’s practical advantages over existing tools can be considered fully established. Donor-level independence of ORAM and GRPT from the training registry was verified on full donor identifiers (0/0/0 shared identifiers pairwise). The proposed implementation has been made publicly available (Zenodo DOI: 10.5281/zenodo.22061923, Section 2.7).

## Figures and Tables

**Table 1:** Distribution of donor records by Human Leukocyte Antigen (HLA) typing resolution, per locus, for the Hellenic Transplant Organization (HTO) training registry and the ORAM (Orama Elpidas Donor Center) and GRPT (Evangelismos Hospital Donor Center) validation cohorts, computed directly from each cohort’s raw typing file. For ORAM and GRPT, typing resolution was determined per donor per locus from the raw DNA typing field: an unresolved second field (“XX” or an NMDP multiple-allele code) was classified as intermediate; a fully numeric second field as high; a serology-only call as low; and no DNA or serology value at that locus as untyped. ORAM and GRPT are overwhelmingly intermediate-resolution (ambiguous two-field) at HLA-A, -B, and -DRB1, and HLA-C and HLA-DQB1 are untyped for the large majority of donors in both cohorts (Section 2.1).

Cohort/Locus	Low (Serologic/1-Field)	Intermediate (2-Field, Ambiguous)	High (2-Field Unambiguous or Better)	Untyped
HTO (*n* = 117,345) HLA-A	0.0%	22.8%	77.0%	0.1%
HTO (*n* = 117,345) HLA-B	0.0%	21.8%	78.1%	0.2%
HTO (*n* = 117,345) HLA-C	0.0%	7.6%	74.8%	17.6%
HTO (*n* = 117,345) HLA-DRB1	0.0%	17.4%	82.6%	0.0%
HTO (*n* = 117,345) HLA-DQB1	0.0%	0.2%	77.0%	22.8%
ORAM (*n* = 20,100) HLA-A	0.0%	86.3%	13.5%	0.1%
ORAM (*n* = 20,100) HLA-B	0.1%	83.6%	16.2%	0.1%
ORAM (*n* = 20,100) HLA-C	0.0%	13.4%	9.6%	77.0%
ORAM (*n* = 20,100) HLA-DRB1	0.1%	51.5%	36.1%	12.2%
ORAM (*n* = 20,100) HLA-DQB1	0.0%	0.8%	7.9%	91.2%
GRPT (*n* = 4353) HLA-A	0.0%	99.8%	0.2%	0.0%
GRPT (*n* = 4353) HLA-B	0.0%	99.7%	0.3%	0.0%
GRPT (*n* = 4353) HLA-C	0.0%	0.7%	0.3%	99.0%
GRPT (*n* = 4353) HLA-DRB1	0.1%	81.2%	1.6%	17.0%
GRPT (*n* = 4353) HLA-DQB1	0.0%	0.0%	0.0%	100.0%

**Table 2:** Resolution categories used to define model inputs (low/intermediate) and prediction targets (high).

Resolution Level	Typical Field Depth	Example (HLA-A)	Common Source
Low (serologic/antigen)	1-field/broad antigen	A2	Legacy serology, older DNA panels
Intermediate	2-field, ambiguous	A*02: AJEXK	Intermediate-resolution SSO/SSP
High	2-field, unambiguous, or higher	A*02:01:01	NGS, high-resolution SBT/SSO

**Table 3:** Summary of performance metrics reported in this article, their definitions, how each is computed, and where results using that metric are reported.

Metric	Definition	How It Is Computed	Where Reported
Per-locus accuracy	Proportion of individuals whose predicted allele pair exactly matches confirmatory typing, at one named locus	Concordant predictions at that locus/all predictions at that locus	Section 3.1; Supplementary Tables S2 and S4
Overall (multilocus) accuracy	Proportion of individuals with an exact match at every predicted locus simultaneously, within one experiment	Joint AND across all predicted loci; necessarily ≤ any single per-locus accuracy	Section 2.3 (defined, not separately tabulated)
Call rate	Proportion of submitted genotypes for which a call was returned at or above a stated Post-P threshold	Calls returned/genotypes submitted; moves opposite to accuracy as the threshold changes	Abstract, Section 3.1
Posterior probability (Post-P)	The model’s confidence in one predicted diplotype, given all diplotypes compatible with the input genotype	Post-P(dt) = L(dt)/sum_i L(d_i), Hardy–Weinberg likelihood L	Section 2.3 and Section 3.1
Top-k concordance	Proportion of individuals for whom the confirmatory genotype is among the k highest-Post-P predictions	Rank predictions by Post-P, descending; check top k (reported for k = 3)	Section 2.7, Supplementary Section S3.4

**Table 4:** Cross-cohort allele vocabulary truncation: the proportion of ORAM and GRPT allele calls, and of their distinct alleles, that fall outside the 99–coverage vocabulary derived from the HTO training registry (Table 5, below), computed on verified donor-independent files (0/0/0 shared donor IDs across HTO/ORAM/GRPT). GRPT columns carry small denominators (4–165 typed calls per locus) because 99.8% of GRPT donors are typed only at ambiguous (“XX”) resolution at HLA-A, which is consistent with GRPT being exactly the kind of low-resolution external cohort the framework is intended to upgrade, but this also makes the GRPT-specific percentages presented here imprecise. The ORAM figures, with thousands of typed calls per locus, are the more reliable indicator: cross-cohort exclusion (18–47% of calls) is far higher than the within-HTO self-consistency estimate in Table 5 (under 1% of calls), indicating a real distributional difference between the ORAM population and the HTO training vocabulary’s common-allele profile. For HLA-DQB1, three of the alleles observed in ORAM had never been seen in HTO at all, whereas every allele observed in GRPT had been, consistent with GRPT’s near-total lack of DQB1 typing (Section 2.1). The GRPT/HLA-DQB1 row illustrates this directly: all four typed calls are ambiguous (“XX”-equivalent), and none resolved to a specific allele, so the calls-outside-vocab percentage is computable (*n* = 4), while the distinct-alleles-outside-vocab denominator is legitimately zero, reported as n/a rather than 0.0% to avoid implying a defined ratio. Accuracy stratified by vocabulary membership. Because a donor whose true allele lies outside the training vocabulary cannot receive a correct two-field call at all, reported accuracy was stratified by whether both of a donor’s confirmatory alleles belong to that vocabulary (Supplementary Table S9). Counting per donor rather than per call, 26–71% of ORAM donors and 0–41% of GRPT donors fall outside it, depending on locus. Within the in-vocabulary stratum, the framework reaches 94.2% first-field and 92.8% two-field accuracy on ORAM (95.8% and 93.4% on GRPT); in the out-of-vocabulary stratum, first-field accuracy falls to 73.7% on ORAM (80.0% on GRPT) and two-field accuracy is zero by construction. The per-locus totals recover the first-field figures reported in Section 3.2, so the headline accuracy should be read as a weighted average over a population in which roughly half of ORAM donors carry at least one allele the model cannot represent at a two-field resolution.

Locus	ORAM Calls Outside Vocab (n Typed)	ORAM Distinct Alleles Outside Vocab	GRPT Calls Outside Vocab (n Typed)	GRPT Distinct Alleles Outside Vocab	Alleles Never Seen in HTO (ORAM/GRPT)
HLA-A	39.8% (16,758)	84.9% (680/801)	20.0% (15)	14.3% (1/7)	210/1
HLA-B	27.9% (17,027)	84.5% (841/995)	14.3% (21)	25.0% (3/12)	85/3
HLA-C	46.5% (8369)	82.9% (189/228)	22.2% (27)	36.4% (4/11)	83/4
HLA-DRB1	24.1% (18,827)	88.6% (263/297)	13.9% (165)	45.5% (15/33)	52/2
HLA-DQB1	17.8% (3200)	69.0% (20/29)	0.0% (4)	n/a (0/0)	3/0

**Table 5:** Allele vocabulary abridgment bias computed directly from the registry dataset (117,345 donor records, unambiguous two-field DNA typing; *n* = 175,546–193,452 allele calls per locus depending on typing completeness). A vocabulary retaining only the alleles needed to cover 99% of observed allele calls excludes the large majority of distinct alleles observed (58–81%, driven by singleton and near-singleton alleles) while excluding under 1% of allele calls, confirming that the abridgment step discards rare-allele diversity much more than it discards typing coverage. Donor level: Among the 86,002 donors (73.3% of this dataset) typed at unambiguous two-field resolution across all five loci, 6.9% carried at least one allele outside this 99–coverage vocabulary at one or more loci.

Locus	Distinct Alleles Observed	Singleton Alleles (% of Distinct)	Alleles Needed for 99% Call Coverage	Distinct Alleles Excluded	Allele Calls Excluded
HLA-A	142	45 (31.7%)	27 (19.0%)	81.0%	0.9%
HLA-B	195	54 (27.7%)	53 (27.2%)	72.8%	0.9%
HLA-C	96	28 (29.2%)	23 (24.0%)	76.0%	0.8%
HLA-DRB1	119	31 (26.1%)	28 (23.5%)	76.5%	0.9%
HLA-DQB1	33	6 (18.2%)	14 (42.4%)	57.6%	0.4%

## Data Availability

The HTO, ORAM, and GRPT donor data are not publicly available due to donor privacy protections and Hellenic Transplant Organization data-sharing restrictions. The simulated reference dataset used for framework-level benchmarking (Supplementary Section S3) was generated from the published, publicly available NMDP US registry haplotype frequency resource of Gragert et al. [8], and it can be regenerated directly from the same public resource following the procedure and the six standard ancestry categories described in Supplementary Section S3.1. A reference implementation, in Python 3.12 and C++17 (compiled with GCC 13.3.0), of the ELM training and inference algorithms, the three ELM extensions, and the comparator algorithms specified in Supplementary Section S2 (Algorithms S1–S5) is open source (MIT License) and available from the repository of the Biomedical Engineering Laboratory, National Technical University of Athens (https://github.com/Biomedical-Engineering-Laboratory-NTUA/hla-elm-toolkit-2026, accessed on 5 October 2026), archived on Zenodo (DOI: 10.5281/zenodo.22061923, accessed on 5 October 2026). This implementation uses a single random hidden layer in place of the domain-structured HL-1/HL-2/HL-3 topology shown in Section 2.4 and is distributed with a synthetic demonstration population, so it documents the algorithms rather than reproducing the figures reported here; the registry-scale production code is bound to the donor data environment described above and is available from the corresponding author on reasonable request.

## Supplementary Materials

The following supporting information can be downloaded at https://www.mdpi.com/article/10.3390/genes17101239/s1. Figure S1: Visual summary of Table 4: cross-cohort allele vocabulary truncation by locus. Figure S2: Visual summary of Table 5: within-registry allele vocabulary abridgment by locus. Figure S3: Top 10 estimated HLA-A~HLA-B haplotype frequencies from EM training on the HTO registry. Figure S4: Visual summary of Table S6: the two-locus EM baseline’s call rate/accuracy trade-off on ORAM (Supplementary Section S4.1). Figure S5: Call rate: ELM (reported) vs. GRIMM on the ORAM and GRPT cohorts. Figure S6: Per-locus first-field accuracy: GRIMM on ORAM and GRPT vs. the ELM framework’s reported range. Figure S7: Call rate and joint accuracy: GRIMM on the HTO training population vs. the ORAM and GRPT cohorts. Figure S8: Call rate: ELM (reported) vs. GRIMM vs. hlaR ImputeHaplo on the GRPT cohort. Figure S9: Per-locus first-field accuracy on GRPT: hlaR ImputeHaplo vs. GRIMM vs. ELM (reported). Figure S10: Call rate: hlaR ImputeHaplo vs. GRIMM vs. ELM (reported) across GRPT, ORAM, and HTO. Figure S11: hlaR ImputeHaplo per-locus first-field accuracy range by cohort (GRPT, ORAM, HTO). Figure S12: Call rate on GRPT: ELM (reported) vs. three haplotype-frequency-based methods. Figure S13: Per-locus first-field accuracy on GRPT: in-house EM baseline vs. hlaR ImputeHaplo vs. GRIMM. Figure S14: Call rate: in-house Hapl-o-Mat-style EM baseline vs. GRIMM vs. ELM (reported) across GRPT, ORAM, and HTO. Figure S15: In-house Hapl-o-Mat-style baseline: per-locus/joint accuracy by cohort (GRPT, ORAM, HTO). Figure S16: Kernel ELM (KELM): the explicit random hidden layer is replaced with an implicit kernel matrix Ω, removing the need to choose a hidden-layer size at the cost of quadratic scaling with training-set size. Figure S17: Weighted ELM (WELM): a diagonal sample-weight matrix W upweights training examples from rare alleles so that the least squares solution does not neglect them relative to common alleles. Algorithm S1: ELM training (resolution upgrading/missing-locus imputation). Algorithm S2: Inference: upgrading a donor genotype to a high-resolution call. Algorithm S3: GRIMM: graph-based five-locus imputation (main text, Section 3.2). Algorithm S4: HaploStats-style haplotype frequency imputation, approximated in this study by the hlaR ImputeHaplo baseline (Section S4.3). Algorithm S5: Hapl-o-Mat: open source EM haplotype frequency estimation and imputation (implemented in this study as the in-house five-locus EM baseline; Section S4.4). Table S1: Composition of the simulated reference dataset. Table S2: Top-1 allele pair concordance. Table S3: Wall-clock training time. Table S4: Top-1 concordance and training time by ELM variant. Table S5: Accuracy on alleles below the locus median frequency. Table S6: Two-locus (HLA-A to HLA-B) EM haplotype frequency baseline. Table S7: GRIMM performance on the HTO training population compared with the external ORAM and GRPT cohorts (Table 10), first-field top-1 concordance (Section S4.2). Table S8: Method comparison across GRPT, ORAM, and HTO: ELM vs. GRIMM (Section 3.2) vs. hlaR ImputeHaplo, an open source HaploStats-inspired baseline (Section S4.3), first-field top-1 concordance. Table S9. Accuracy stratified by allele vocabulary membership, per locus, and cohort.

## References

[1] Shaw B.E., Logan B.R., Spellman S.R., Marsh S.G., Robinson J., Pidala J., Hurley C., Barker J., Maiers M., Dehn J. (2018). Development of an unrelated donor selection score predictive of survival after HCT: Donor age matters most. Biol. Blood Marrow Transplant..

[2] Geo J.A., Ameen R., Al Shemmari S., Thomas J. (2024). Advancements in HLA typing techniques and their impact on transplantation medicine. Med. Princ. Pract..

[3] Wittig M., Anmarkrud J.A., Kässens J.C., Koch S., Forster M., Ellinghaus E., Hov J.R., Sauer S., Schimmler M., Ziemann M. (2015). Rapid, scalable and highly automated HLA genotyping using next-generation sequencing: A transition from research to diagnostics. Nucleic Acids Res..

[4] Yin Y., Lan J.H., Nguyen D., Valenzuela N., Takemura P., Bolon Y.-T., Springer B., Saito K., Zheng Y., Hague T. (2016). Application of high-throughput next-generation sequencing for HLA typing on buccal extracted DNA: Results from over 10,000 donor recruitment samples. PLoS ONE.

[5] Kogler G., Enczmann J., Rocha V., Gluckman E., Wernet P. (2005). High-resolution HLA typing by sequencing for HLA-A, -B, -C, -DR, -DQ in unrelated cord blood/patient pairs. Bone Marrow Transplant..

[6] Howell W.M., Carter V., Clark B. (2010). The HLA system: Immunobiology, HLA typing, antibody screening and crossmatching techniques. J. Clin. Pathol..

[7] Excoffier L., Slatkin M. (1995). Maximum-likelihood estimation of molecular haplotype frequencies in a diploid population. Mol. Biol. Evol..

[8] Gragert L., Madbouly A., Freeman J., Maiers M. (2013). Six-locus high resolution HLA haplotype frequencies derived from mixed-resolution DNA typing for the entire US donor registry. Hum. Immunol..

[9] Madbouly A., Gragert L., Freeman J., Leahy N., Gourraud P., Hollenbach J.A., Kamoun M., Fernandez-Vina M., Maiers M. (2014). Validation of statistical imputation of allele-level multilocus phased genotypes from ambiguous HLA assignments. Tissue Antigens.

[10] National Marrow Donor Program HaploStats: Haplotype-Based HLA Imputation. https://haplostats.org.

[11] Maiers M., Halagan M., Gragert L., Bashyal P., Brelsford J., Schneider J., Lutsker P., Louzoun Y. (2019). GRIMM: GRaph IMputation and matching for HLA genotypes. Bioinformatics.

[12] Schaefer C., Sauter J., Schmidt A.H. (2017). Hapl-o-Mat: Open-source software for HLA haplotype frequency estimation from ambiguous and heterogeneous typing data. BMC Bioinform..

[13] Zheng X., Shen J., Cox C., Wakefield J.C., Ehm M.G., Nelson M.R., Weir B.S. (2014). HIBAG—HLA genotype imputation with attribute bagging. Pharmacogenom. J..

[14] Dilthey A.T., Moutsianas L., Leslie S., McVean G. (2011). HLA*IMP—An integrated framework for imputing classical HLA alleles from SNP genotypes. Bioinformatics.

[15] Engen R.M., Jedraszko A.M., Conciatori M.A., Tambur A.R. (2021). Substituting imputation of HLA antigens for high-resolution HLA typing: Evaluation of a multiethnic population and implications for clinical decision making in transplantation. Am. J. Transplant..

[16] Lhotte R., Letort V., Usureau C., Jorge-Cordeiro D., PHRC Acorghla Consortium, Siemowski J., Gabet L., Cournède P.-H., Taupin J.-L. (2024). Improving HLA typing imputation accuracy and eplet identification with local next-generation sequencing training data. HLA.

[17] Rumelhart D.E., Hinton G.E., Williams R.J. (1986). Learning representations by back-propagating errors. Nature.

[18] Huang G.B., Zhu Q.Y., Siew C.K. (2006). Extreme learning machine: Theory and applications. Neurocomputing.

[19] Huang G.B., Wang D.H., Lan Y. (2011). Extreme learning machines: A survey. Int. J. Mach. Learn. Cybern..

[20] Huang G.B. (2014). An insight into extreme learning machines: Random neurons, random features and kernels. Cogn. Comput..

[21] Ding S., Zhao H., Zhang Y., Xu X., Nie R. (2015). Extreme learning machine: Algorithm, theory and applications. Artif. Intell. Rev..

[22] Marsh S.G.E., Albert E.D., Bodmer W.F., Bontrop R.E., Dupont B., Erlich H.A., Geraghty D.E., Holdsworth R., Hurley C.K., Lau M. (2010). Nomenclature for factors of the HLA system, 2010. Tissue Antigens.

[23] Robinson J., Barker D.J., Georgiou X., Cooper M.A., Flicek P., Marsh S.G.E. (2020). IPD-IMGT/HLA Database. Nucleic Acids Res..

[24] Hardy G.H. (1908). Mendelian proportions in a mixed population. Science.

[25] Huang G.B., Zhou H., Ding X., Zhang R. (2012). Extreme learning machine for regression and multiclass classification. IEEE Trans. Syst. Man Cybern. B Cybern..

[26] Zong W., Huang G.B., Chen Y. (2013). Weighted extreme learning machine for imbalance learning. Neurocomputing.

[27] Zhang Y., Liu B., Cai J., Zhang S. (2017). Ensemble weighted extreme learning machine for imbalanced data classification based on differential evolution. Neural Comput. Appl..

[28] Friedman J.H. (2001). Greedy function approximation: A gradient boosting machine. Ann. Stat..

[29] Chen T., Guestrin C. (2016). XGBoost: A scalable tree boosting system. Proceedings of the 22nd ACM SIGKDD International Conference on Knowledge Discovery and Data Mining.

[30] Breiman L. (2001). Random forests. Mach. Learn..

[31] Kohavi R. (1995). A study of cross-validation and bootstrap for accuracy estimation and model selection. Proceedings of the 14th International Joint Conference on Artificial Intelligence.

[32] Hastie T., Tibshirani R., Friedman J. (2009). The Elements of Statistical Learning: Data Mining, Inference, and Prediction.

[33] Efron B. (1979). Bootstrap methods: Another look at the jackknife. Ann. Stat..

[34] Wilson E.B. (1927). Probable inference, the law of succession, and statistical inference. J. Am. Stat. Assoc..

[35] Zhang J., Johnson A., Larsen C.P. (2023). hlaR: Tools for HLA Data, R Package Version 1.0.0. https://cran.r-project.org/web/packages/hlaR/index.html.

[36] Liang N.Y., Huang G.B., Saratchandran P., Sundararajan N. (2006). A fast and accurate online sequential learning algorithm for feedforward networks. IEEE Trans. Neural Netw..

[37] Weimer E.T., Newhall K.A. (2025). Machine learning enhanced immunologic risk assessments for solid organ transplantation. Sci. Rep..

