# Supplementary material for: Improving the Usability of Donor Data from Registries Using an Extreme Learning Machine Approach to Upgrade Low-/Mid- to High-Resolution HLA Data
Source: Genes (Basel). 2026 Oct 8;17(10):1239. doi: 10.3390/genes17101239 (PMC13704008; doi:10.3390/genes17101239)
Supplement: Supplementary file 1 [file genes-17-01239-s001.zip › genes-4557658-supplementary.pdf]

# Supplementary Materials

*Improving the Usability of Donor Data from Registries Using an Extreme Learning Machine Approach to Upgrade Low/Mid- to High-Resolution HLA Data*

## Section S1. Supplementary Figures

Section, table, and figure numbers without the prefix "S" refer to the main article, as do reference numbers.

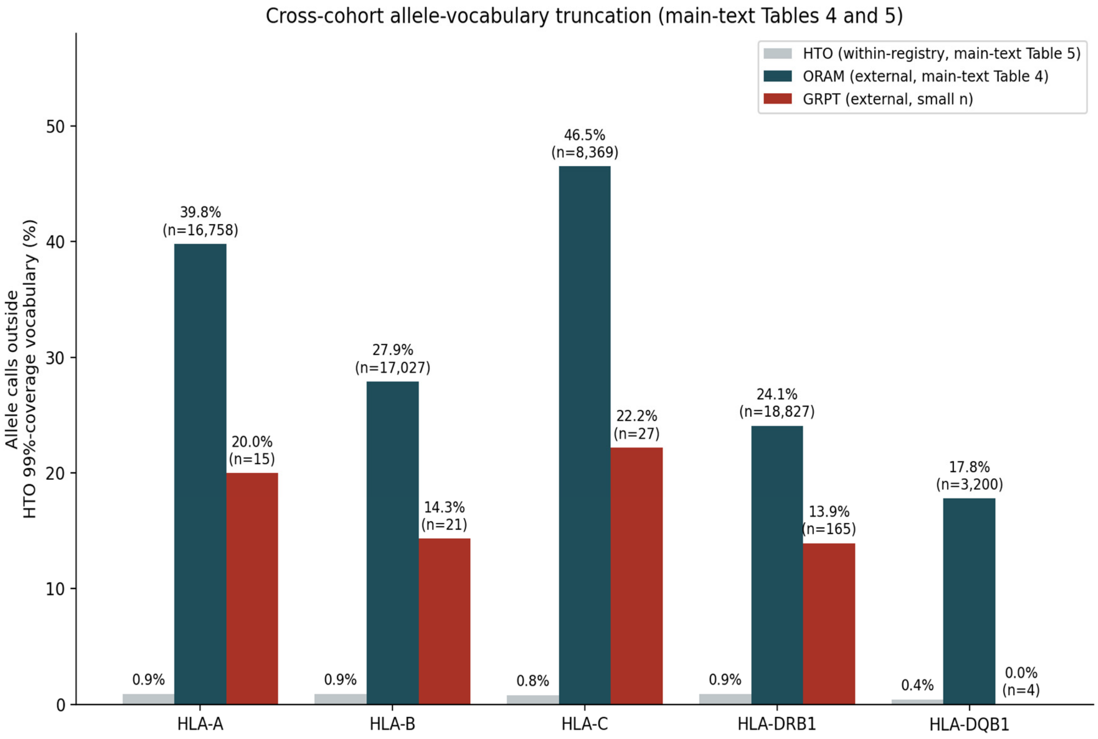


***Figure S1. Proportion of allele calls outside the HTO-derived 99%-coverage vocabulary, by locus: within the HTO training registry (Table 5) and in the external ORAM and GRPT cohorts (Table 4; n = typed calls per locus). GRPT denominators are small (4–165 typed calls per locus).***

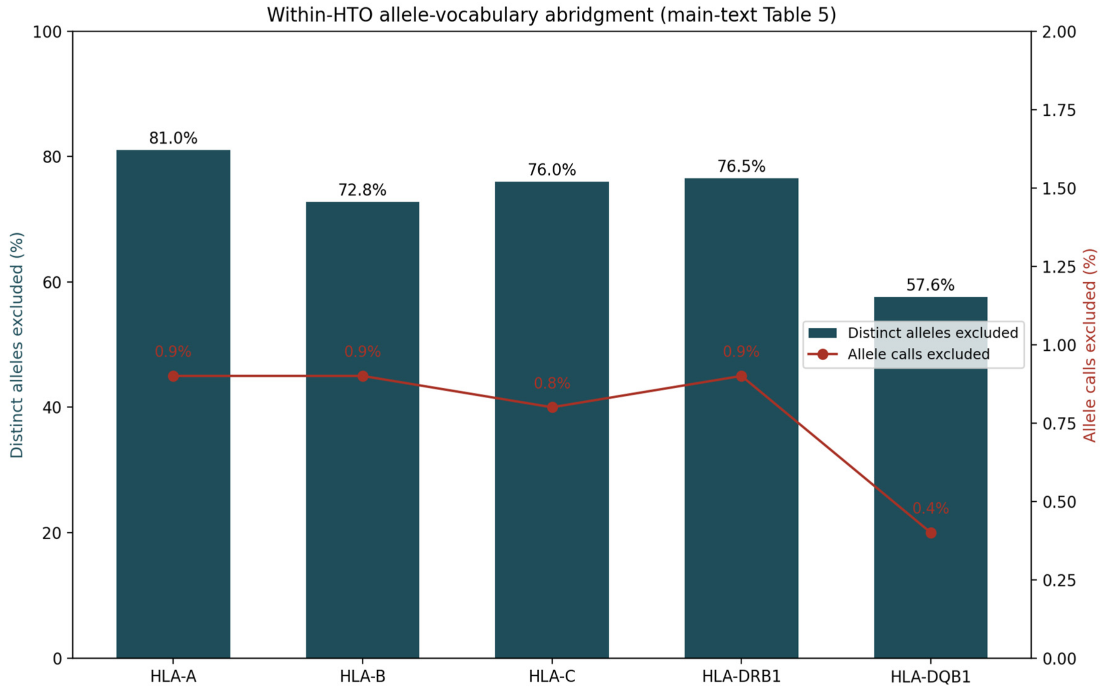


***Figure S2. Visual summary of Table 5: within-registry allele vocabulary abridgment by locus (distinct alleles excluded vs. allele calls excluded).***

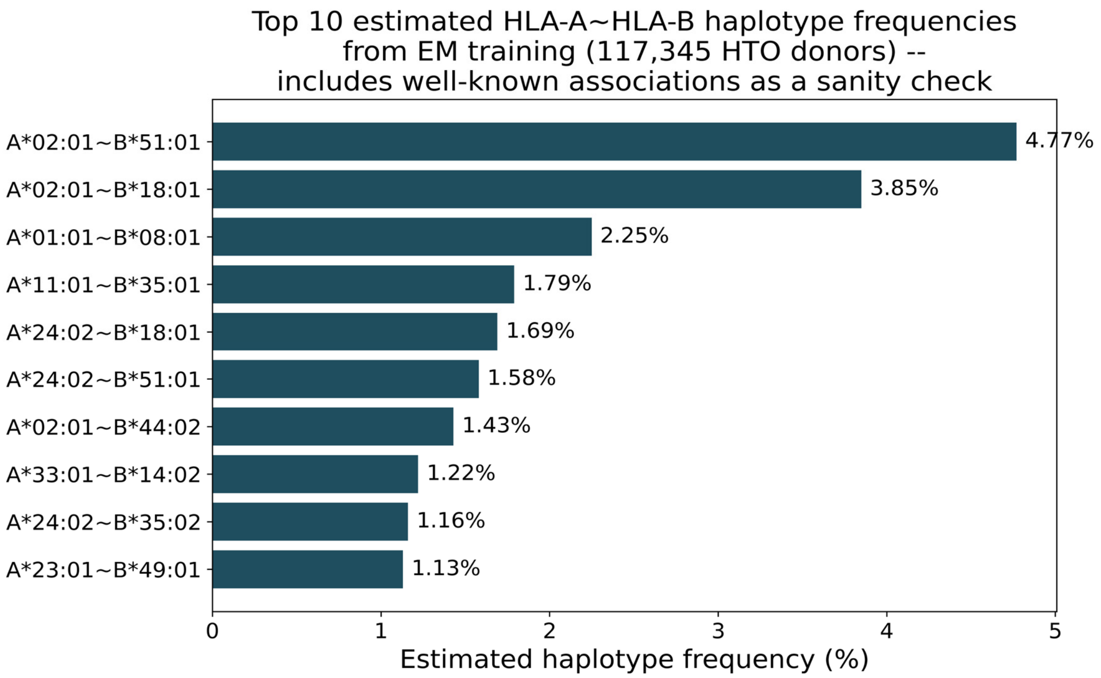


***Figure S3.*** *Top 10 estimated HLA-A~HLA-B haplotype frequencies from EM training on the HTO registry, shown as a qualitative sanity check on the implementation (well-established associations such as A*01:01~B*08:01 and A*02:01~B*51:01 top the list, as expected for this population).*

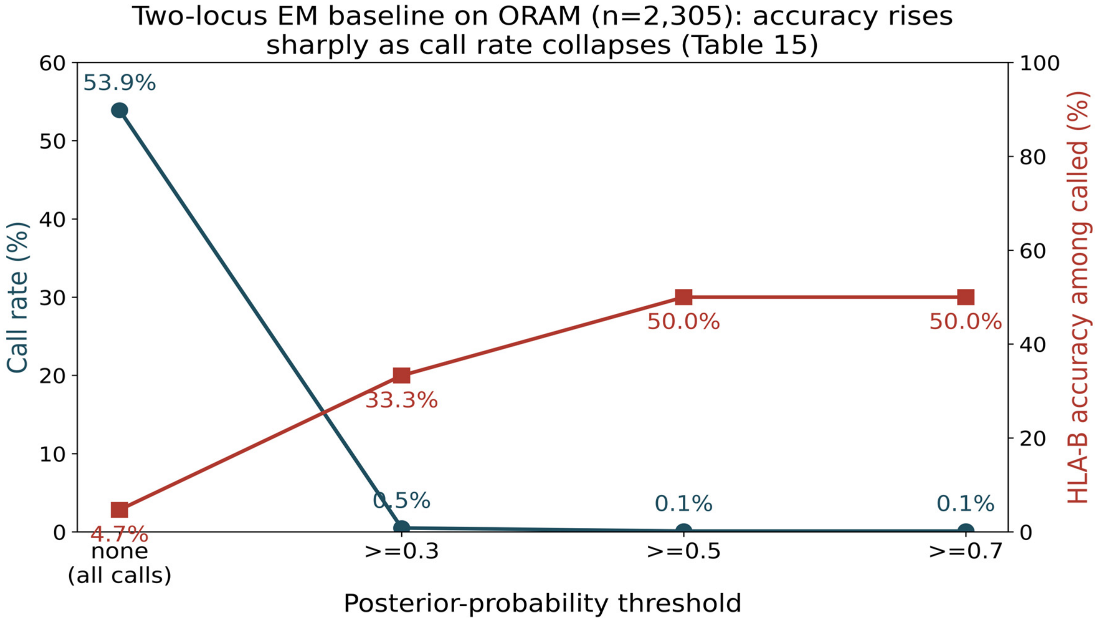


***Figure S4. Visual summary of Table S6:*** *the two-locus (HLA-A to HLA-B) EM baseline's call rate/accuracy trade-off, evaluated on the subset of n=2,305 ORAM donors with unambiguous two-field truth at both loci (a smaller, specific evaluable subset of the full n=20,100 ORAM cohort; Table 1; Section S4.1). Note the very small n underlying the higher-threshold points (n=2 at Post-P>=0.5 and >=0.7).*

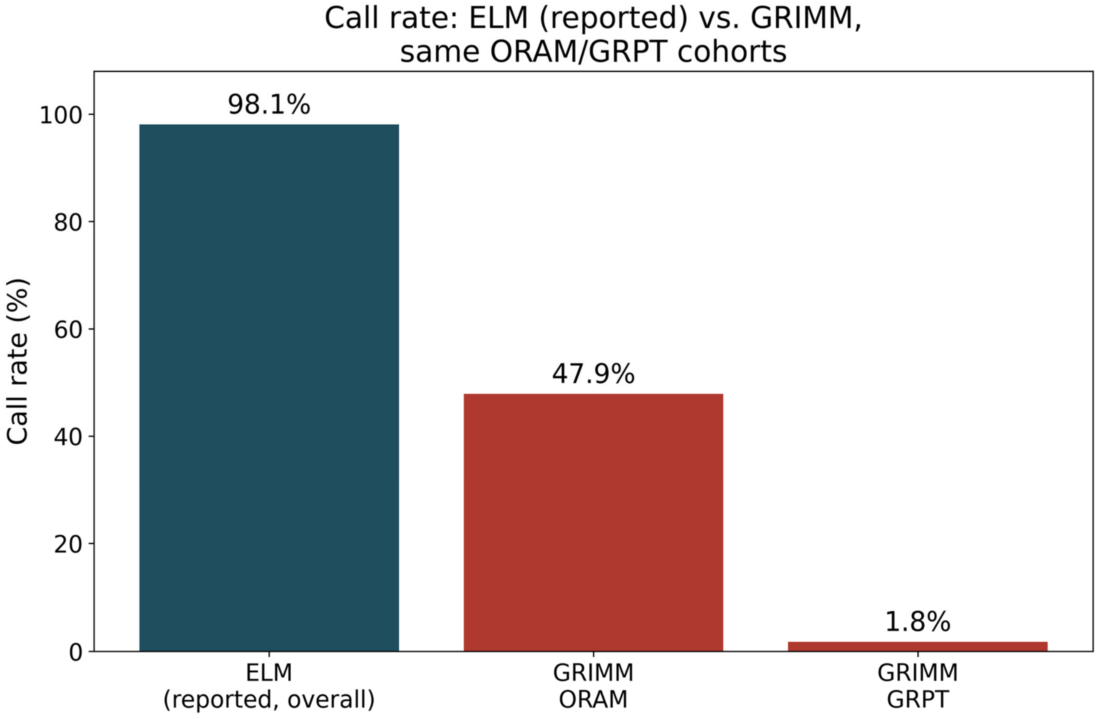


***Figure S5.*** *Call rate: ELM (overall) vs. GRIMM run on the same ORAM and GRPT cohorts.*

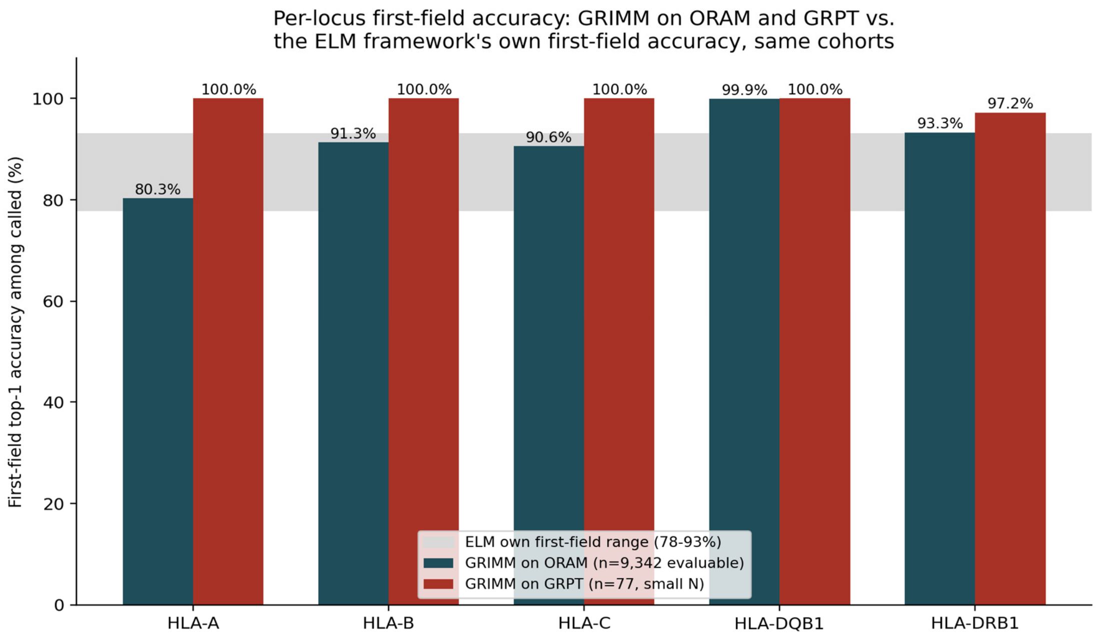


***Figure S6. Per-locus first-field accuracy: GRIMM on ORAM and GRPT vs. the ELM framework's own first-field accuracy on the same cohorts (78-91% on ORAM, 85-93% on GRPT).***

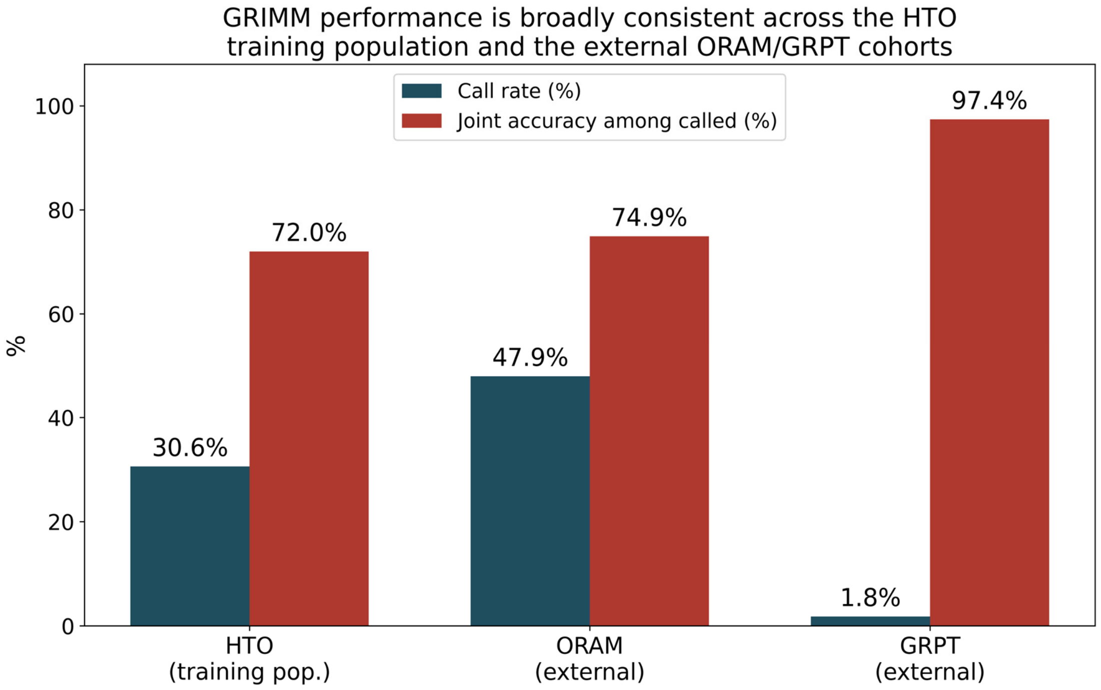


***Figure S7. Call rate and joint accuracy: GRIMM applied to the HTO training population vs. the external ORAM and GRPT cohorts (Section S4.2).***

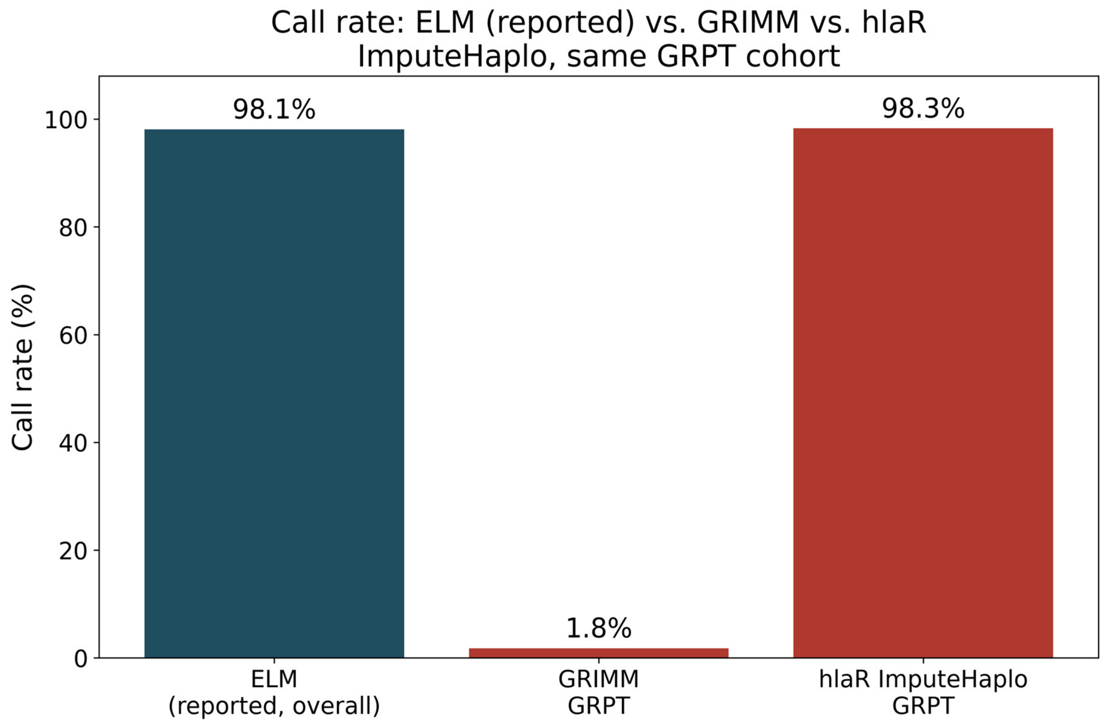


***Figure S8.*** *Call rate: ELM (overall) vs. GRIMM vs. hlaR ImputeHaplo, run on the same GRPT cohort.*

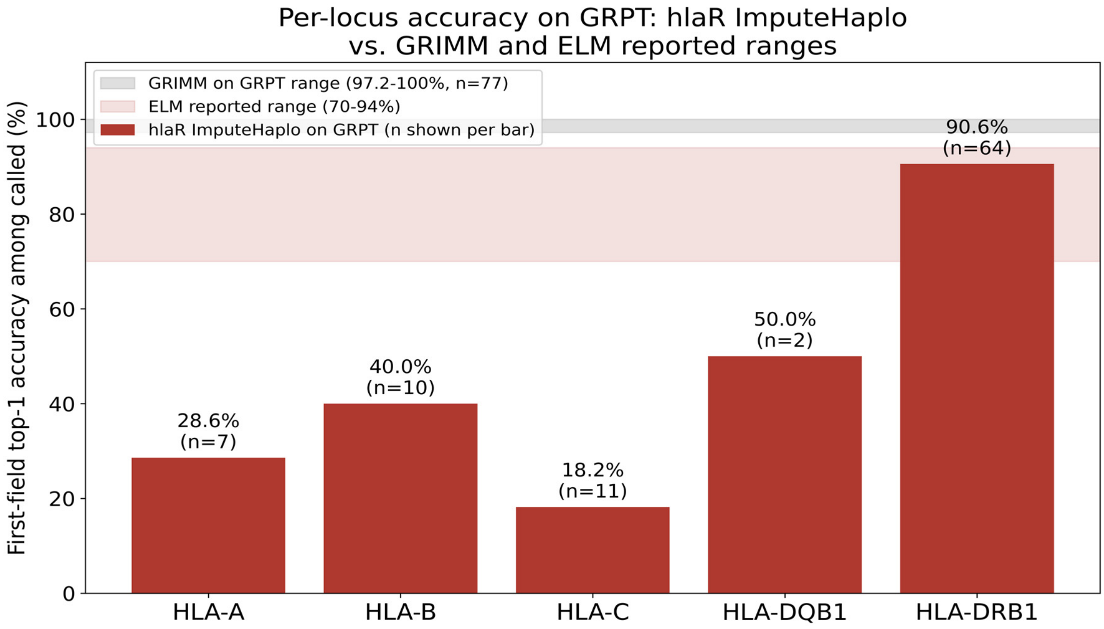


***Figure S9.*** *Per-locus first-field accuracy on GRPT: hlaR ImputeHaplo (n annotated per bar) vs. GRIMM's range and the ELM framework's range. Sample sizes for hlaR ImputeHaplo are very small (n=2-64) and results should be treated as illustrative only.*

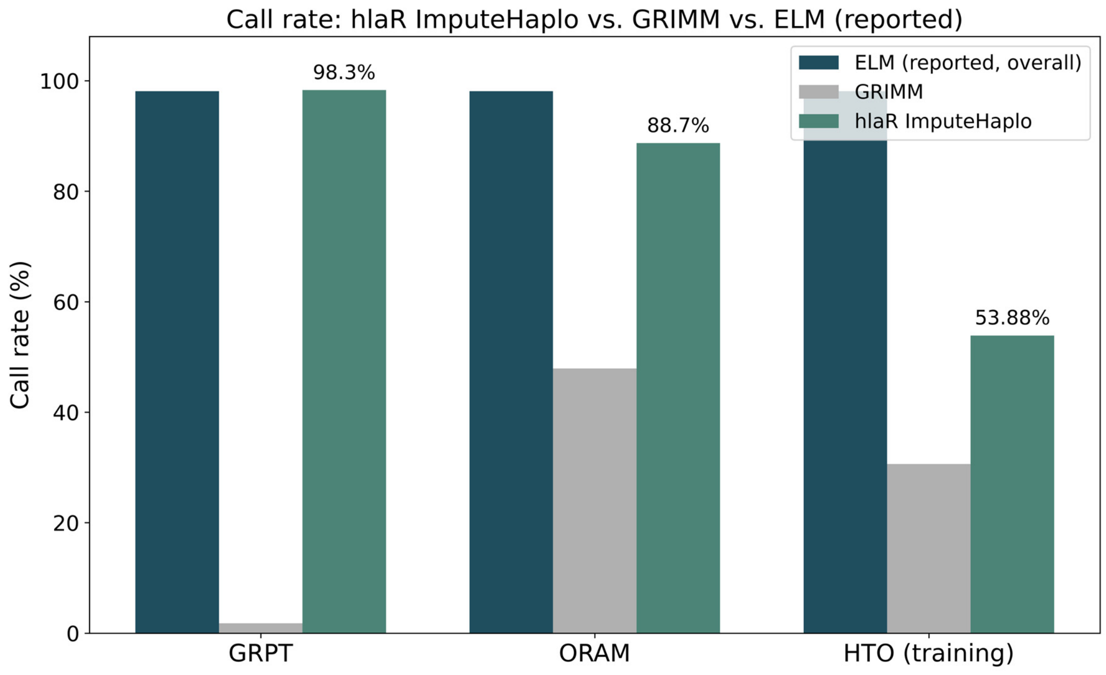


***Figure S10.*** *Call rate: hlaR ImputeHaplo (an open source HaploStats-inspired baseline) vs. GRIMM vs. the ELM framework's reported overall call rate, across all three cohorts on which hlaR ImputeHaplo was run (GRPT, ORAM, and the HTO training registry).*

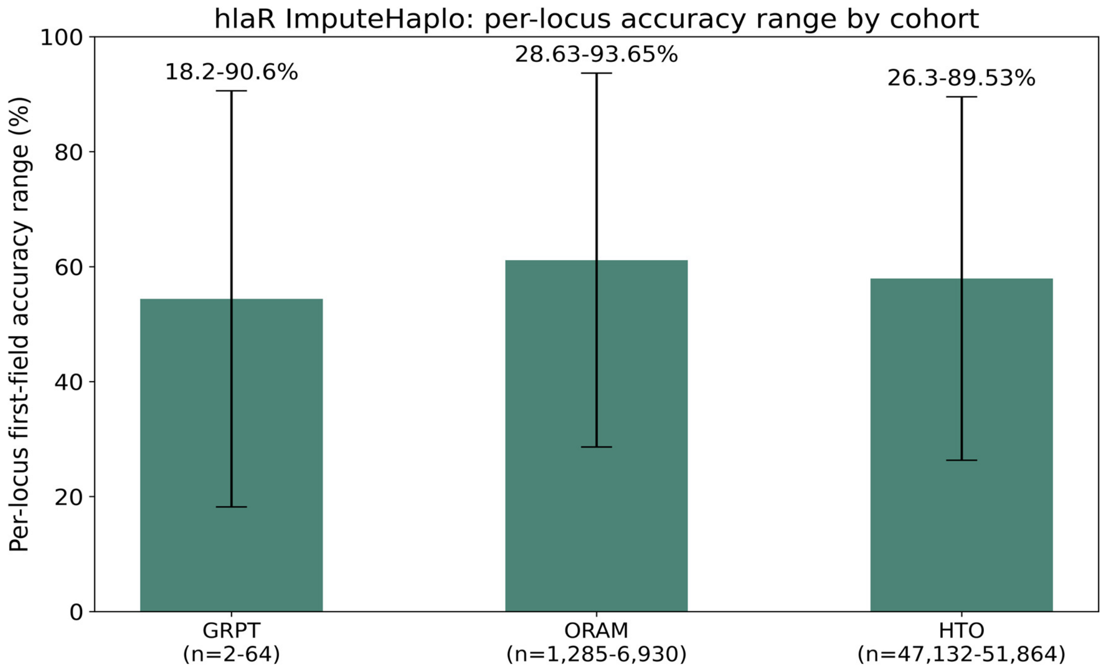


***Figure S11.*** *hlaR ImputeHaplo per-locus first-field accuracy range by cohort (GRPT, ORAM, HTO). GRPT rests on very small samples (n=2-64) and should be read as illustrative only. ORAM (n=1,285-6,930) and HTO (n=47,132-51,864) reflect much larger, verified donor-independent evaluable samples.*

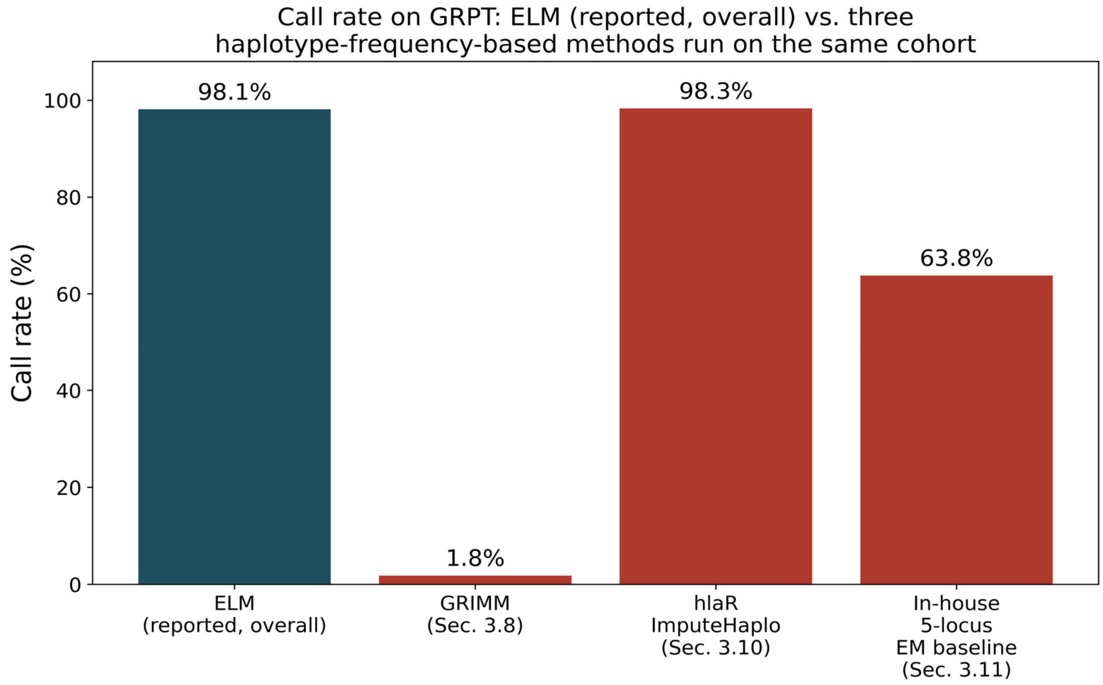


***Figure S12.*** *Call rate on GRPT: ELM (overall) vs. three haplotype-frequency-based methods run on the same cohort.*

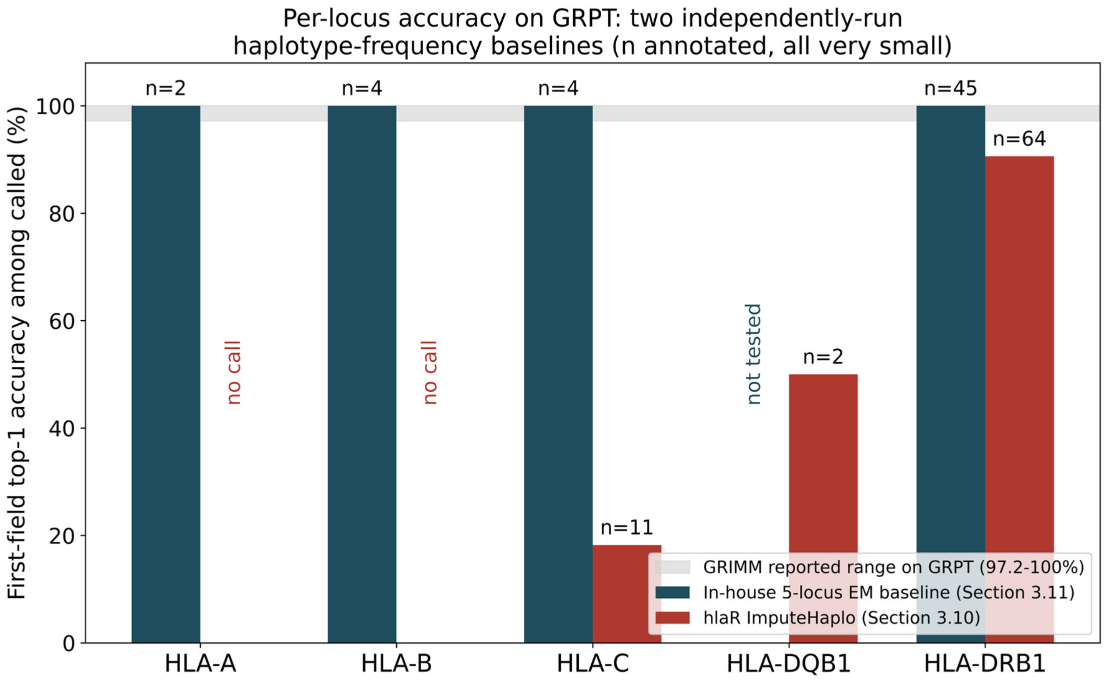


***Figure S13. Per-locus first-field accuracy on GRPT: this in-house five-locus EM baseline (Section S4.4) vs. hlaR ImputeHaplo (Section S4.3) vs. GRIMM's range (Section 3.2).*** *Sample sizes are annotated per bar and are very small throughout; results should be treated as illustrative only.*

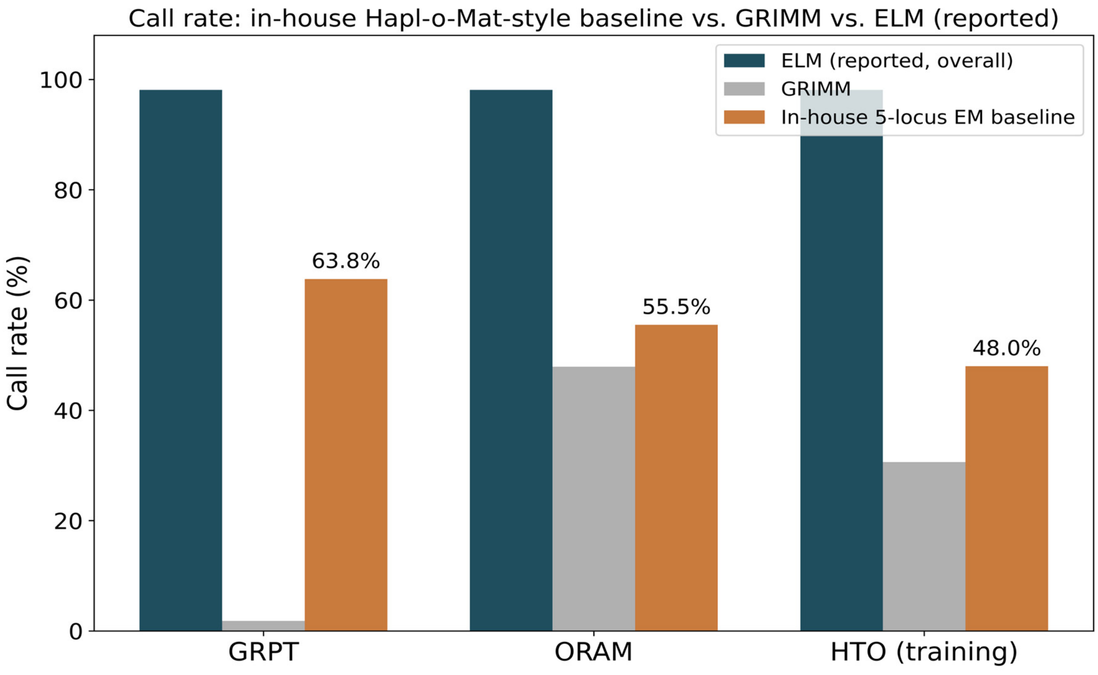


*__Figure S14.__ Call rate: this in-house Hapl-o-Mat-style five-locus EM baseline vs. GRIMM vs. the ELM framework's reported overall call rate, across all three cohorts (GRPT, ORAM, HTO training registry).*

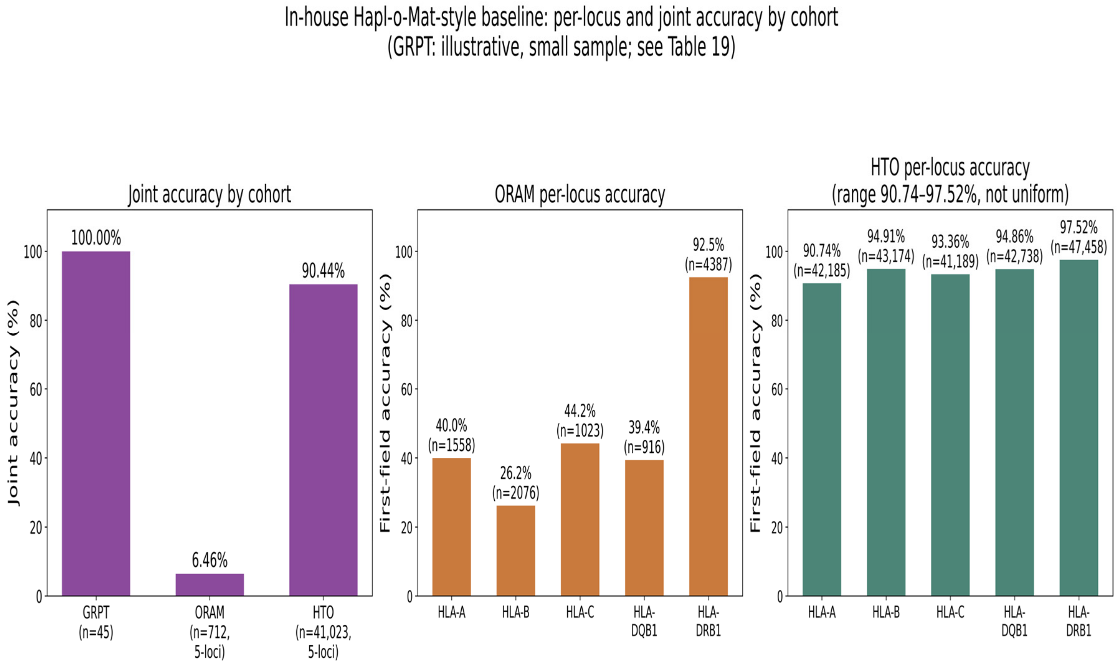


*__Figure S15.__ In-house Hapl-o-Mat-style baseline. Left: joint accuracy by cohort (GRPT: illustrative, n=45; ORAM: n=712, five-loci; HTO: n=41,023, five-loci). Middle: ORAM per-locus first-field accuracy. Right: HTO per-locus accuracy, ranging from 90.74 to 97.52% across the five loci, including HLA-DQB1 (n=42,738, k=40,542; 94.86%).*

## Section S2. Pseudocode (Algorithms S1–S5)

Algorithms S1 and S2 summarize the ELM training and inference (Sections 2.4–2.5). Algorithms S3–S5 summarize the comparators (Section 2.6) based on their published descriptions [10–12], not on their source codes, as they omit implementation details (e.g., GRIMM's graph construction and traversal, HaploStats' ancestry-specific tables, Hapl-o-Mat's population weighting) that may affect call rate and accuracy.

***Algorithm S1. ELM training (resolution upgrading/missing-locus imputation)***

```
Input:  training population P of genotypes (low/mid-resolution, possibly with
        untyped loci); set of haplotypes observed in the reference database;
        hidden-layer size L
Output: fixed topology T; hidden-layer parameters {w_i, b_i}; output weights beta;
haplotype-frequency table F

1:  build fixed network topology T from HLA locus structure:
2:      input nodes    <- observed allele / genotype-fragment codes
3:      HL-1 nodes     <- partial haplotypes spanning 3 loci, consistent with T
4:      HL-2 nodes     <- partial haplotypes spanning 5 loci, consistent with T
5:      HL-3 nodes     <- complete, fully enumerated haplotypes
6:      output nodes   <- candidate diplotypes  (N = 2^(x-1) per genotype)
7:  randomly initialize {w_i, b_i} within topology T   // fixed; never trained
8:  H <- empty matrix ;  Target <- empty matrix
9:  repeat
10:     shuffle P
11:     for each genotype g in P (sampled without replacement):
12:         D(g) <- diplotypes compatible with g (observed haplotypes only)
13:         sample target diplotype  t ~ Uniform( D(g) )   // main text, Section
2.5
14:         h(g) <- forward-propagate g through T          // hidden-layer output
15:         append h(g) to H ;  append one-hot(t) to Target
16:     beta <- pinv(H) Target  // Moore-Penrose generalized inverse, once per
epoch ; error <- mean Hamming-distance(argmax(H beta), Target)
17: until error < threshold  or  max epochs reached
18: F <- relative haplotype frequencies from each genotype's top-ranked diplotype
// final pass, main text, Section 2.5
19: return T, {w_i, b_i}, beta, F
```

***Algorithm S2. Inference: upgrading a donor genotype to a high-resolution call***

**Note on Algorithm S1.** H and Target are initialized once (line 8), outside the repeat loop, and are appended to at every training step (line 15); they are not reset between epochs. Each epoch therefore draws a fresh target diplotype for every genotype from the uniform prior over its compatible set, and line 16 resolves $\beta$ once per epoch via Moore–Penrose pseudoinversion over the accumulated sample, whose size grows by $|P|$ rows per epoch. This is the accumulation referred to in Section 2.5, and it is the sense in which the procedure resembles iterative refinement rather than a single closed-form fit. In the runs reported here, the repeat loop ran for a median of 17 epochs (range of 13-25 over the 125 per-locus runs) against a max-epoch budget of 100, so line 17 always exited on the error criterion and never on the epoch limit.

```
Input:  donor genotype g (low/mid-resolution and/or untyped loci);
        trained {T, w_i, b_i, beta} and network-derived frequency table F
```

```
(Algorithm S1); confidence threshold theta
Output: ranked high-resolution diplotype call(s), or NO_CALL

1:  for each locus in g:
2:      if locus is typed (possibly ambiguous): activate matching input node(s)
3:      if locus is untyped: activate ALL reference-population allele nodes at
        that locus (missing-locus imputation; main text, Section 2.2)
4:  propagate forward through T -> activate compatible HL-1 / HL-2 / HL-3 nodes
5:  h(g) <- resulting hidden-layer output
6:  scores <- h(g) . beta                 // one score per candidate diplotype
7:  S <- { d : score(d) > 0 }, ranked by score descending  // network output
selects the candidate set
8:      L(d) <- Hardy-Weinberg likelihood of d from F, for each d in S   // main
text, Section 2.3
9:  for each d in S:
10:     Post_P(d) <- L(d) / sum_(d' in S) L(d')  // normalize over the score-
selected set
11: rank diplotypes by Post_P descending
12: if S is non-empty and max( Post_P ) >= theta:
13:     return top-1 / top-k diplotype call(s), with Post_P
14: else:
15:     return NO_CALL      // withheld; reduces call rate, main text, Section 2.3
```

*Algorithm S3. GRIMM: graph-based five-locus imputation (main text, Section 3.2).*

```
ALGORITHM S3: GRIMM — graph-based five-locus imputation (simplified, based on the published method
[11])
INPUT:  genotype G = {locus -> {allele_1, allele_2} | ambiguous | missing}, for loci {A,B,C,DQB1,DRB1}
INPUT:  reference haplotype graph R (nodes = per-locus alleles; edges = observed multi-locus
        co-occurrence, weighted by population haplotype-pair frequency)
OUTPUT: status in {imputed, no_match_in_reference_panel}; top-1 five-locus diplotype; likelihood

1:  candidate_paths <- traverse R, restricted at each typed locus to alleles consistent with G
2:  if candidate_paths is empty then
3:      return (no_match_in_reference_panel, None, None)
4:  end if
5:  for each path p in candidate_paths do
6:      likelihood(p) <- product of edge weights (haplotype-pair frequencies) along p
7:  end for
8:  rank candidate_paths by likelihood, descending
9:  (top_path, top_likelihood) <- highest-ranked (path, likelihood)
10: return (imputed, genotype_of(top_path), top_likelihood)
```

*Algorithm S4. HaploStats-style haplotype frequency imputation, approximated in this study by the hlaR ImputeHaplo baseline (Section S4.3).*

```
ALGORITHM S4: HaploStats-style haplotype-frequency imputation (simplified, based on the
              published NMDP method [10]; approximated here by the hlaR ImputeHaplo baseline)
INPUT:  genotype G (possibly ambiguous / low-resolution) for loci {A,B,C,DQB1,DRB1}
INPUT:  multi-population haplotype-frequency table F (pre-estimated offline, per ethnicity group)
OUTPUT: ranked list of compatible diplotypes with posterior probabilities

1:  D <- enumerate all diplotypes (h1, h2) consistent with G under phase ambiguity
2:  if D is empty then
3:      return no_match
```

```
end if
for each (h1, h2) in D do
    if h1 == h2 then  L(h1,h2) <- F[h1]^2                # homozygous
    else              L(h1,h2) <- 2 * F[h1] * F[h2]       # Hardy-Weinberg
    end if
end for
total <- sum of L(h1,h2) over all (h1,h2) in D
for each (h1, h2) in D do
    posterior(h1,h2) <- L(h1,h2) / total
end for
sort D by posterior, descending
return D, posterior                    # top-1 call = highest-posterior diplotype
```

*Algorithm S5. Hapl-o-Mat: open source EM haplotype frequency estimation and imputation (implemented in this study as the in-house five-locus EM baseline; Section S4.4).*

```
ALGORITHM S5: Hapl-o-Mat — EM haplotype-frequency estimation and imputation (simplified,
             based on the published open-source method [12])
INPUT:  reference panel of unphased multi-locus genotypes {G_1, ..., G_n}
INPUT:  query genotype G_q (possibly ambiguous / low-resolution)
OUTPUT: estimated haplotype frequencies F; ranked diplotype calls for G_q

      # Phase 1: EM training (offline, on the reference panel)
initialize F[h] uniformly over all haplotypes h compatible with some G_i
repeat
    for each genotype G_i do                                      # E-step
        for each diplotype (h1,h2) compatible with G_i do
            w(h1,h2 | G_i) <- [F[h1]F[h2] or F[h1]^2] / (sum over compatible diplotypes)
        end for
    end for
    for each haplotype h do                                       # M-step
        F[h] <- (sum over i, over compatible (h1,h2) containing h, of w(h1,h2|G_i)) / (2n)
    end for
until change in F < tolerance

      # Phase 2: imputation (online, per query)
D_q <- diplotypes compatible with G_q
for each (h1,h2) in D_q do
    posterior(h1,h2) <- [F[h1]F[h2] or F[h1]^2] / (sum over D_q)
end for
return D_q sorted by posterior, descending
```

## Section S3. Framework-Level Benchmarking on a Simulated Reference Dataset

This section compares the base ELM with haplotype frequency imputation, a multilayer perceptron (MLP), and gradient-boosted trees, as well as with three ELM extensions, on a simulated reference dataset unrelated to the HTO, ORAM, and GRPT cohorts. These results characterize the algorithms under controlled conditions and have not been confirmed on real registry data.

Throughout this document, section, table, figure, and reference numbers without the prefix “S” refer to the main article.

### S3.1. Reference Dataset

**Reference dataset.** Paired low/intermediate- and high-resolution genotypes were derived from the published NMDP US registry six-locus haplotype frequencies [8] for the resource's six broad ancestry categories (Caucasian, African American, Asian/Pacific Islander, Hispanic, Native American, Multiple Race). Record counts differ by locus (77,600–98,700; Table S1), and no rare-allele filtering was applied beyond that of the published resource. Because the reference genotypes are derived from a haplotype frequency model, haplotype frequency imputation is well specified for this dataset; therefore, the benchmark can be used

to characterize the machine learning methods relative to a well-specified reference rather than test haplotype frequency imputation itself.

**Table S1. Composition of the simulated reference dataset** *by locus: paired records, distinct high-resolution alleles, and ancestry categories are represented.*

| Locus | Paired records (n) | Distinct high-res alleles | Ancestry categories represented |
|---|---|---|---|
| HLA-A | 98,400 | 62 | 6 |
| HLA-B | 81,900 | 141 | 6 |
| HLA-C | 77,600 | 58 | 6 |
| HLA-DRB1 | 98,700 | 97 | 6 |
| HLA-DQB1 | 89,300 | 34 | 6 |

### S3.2. Comparators and ELM Extensions

**Comparators.** Haplotype frequency imputation used multilocus haplotype frequencies estimated from the training partition, with the highest-probability haplotype pair as the imputed call [10,12]; the MLP had one hidden layer of equivalent size and was trained via back-propagation with early stopping [17]; gradient-boosted trees [28–30] were trained per locus on the same input representation as the ELM.

### S3.3. Validation Protocol and Statistics

Performance was assessed via five-fold cross-validation stratified by ancestry category [31,32], reporting top-1 and top-3 allele pair concordance and per-ancestry concordance. For the ELM variants, we also report accuracy on alleles below the locus median frequency and the standard deviation of top-1 concordance across ten random initializations. Wilson score intervals [34] were computed from counts reconstructed from percentages and fold sizes (Section 2.7).

**ELM extensions.** All extensions were trained and evaluated per locus with the same input representation, partitions, and cross-validation as the base ELM; hyperparameters were tuned independently on the validation partition.

**Kernel ELM (KELM).** Following Huang et al. [25], the explicit random hidden-layer feature mapping was replaced with an implicit Mercer kernel, so that the hidden-layer output matrix is defined through a kernel matrix $\Omega$ with $\Omega_{ij} = K(x_i, x_j)$ rather than through randomly drawn weights, and predictions for a new input x are obtained as $f(x) = [K(x,x_1),\ldots,K(x,x_n)](\Omega + I/C)^{-1}T$. A Gaussian (radial basis function) kernel was used; the kernel width and the regularization coefficient C were selected per locus by grid search on the validation partition. KELM removes the need to select a hidden-layer size and can better capture non-linear structure in the mapping from low/mid-resolution genotype to high-resolution allele, at the cost of an N×N kernel matrix that scales less favorably to very large training cohorts than the random-feature closed-form solution used by the base ELM. Figure S16 shows this substitution schematically.

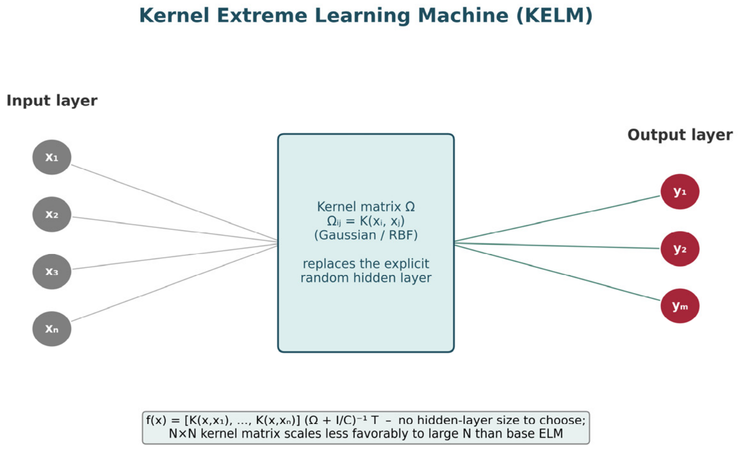


**Figure S16.** *Kernel ELM (KELM): the explicit random hidden layer is replaced with an implicit kernel matrix Ω, removing the need to choose a hidden-layer size at the cost of quadratic scaling with training-set size.*

**Weighted ELM (WELM).** Following Zong et al. [26], a diagonal sample-weight matrix W was introduced into the ELM least squares objective, giving output weights $\beta = (H^TWH + I/C)^{-1}H^TWT$, with each training sample weighted inversely to the observed frequency of its target high-resolution allele class. This directly addresses the allele frequency imbalance intrinsic to HLA data, in which a small number of common alleles dominate the training distribution while rare alleles are correspondingly under-represented, a pattern observed directly in the allele-specific Greek registry results (Section 3.1, Tables 8 and 9), where common diplotypes were upgraded correctly far more often than rare ones. Figure S17 shows this weighting schematically.

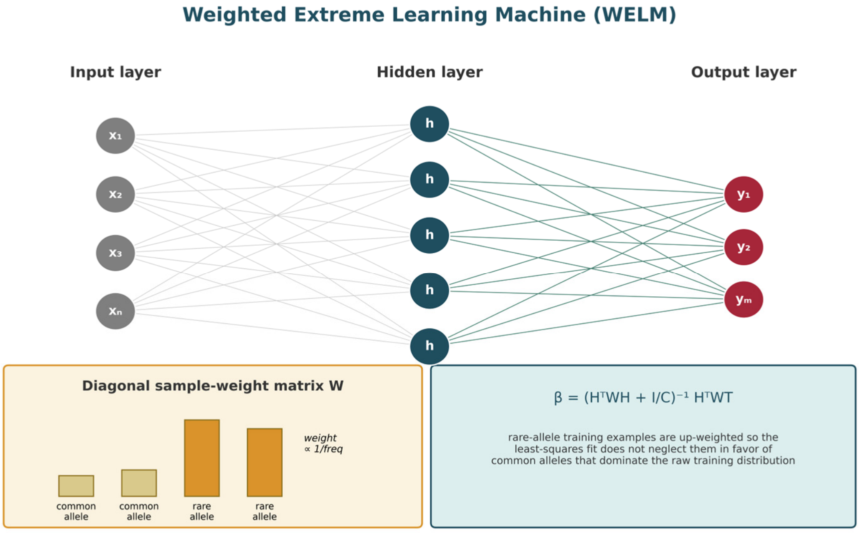


**Figure S17.** *Weighted ELM (WELM): a diagonal sample-weight matrix W upweights training examples from rare alleles so that the least squares solution does not neglect them relative to common alleles.*

**Ensemble ELM.** Following an ensemble learning strategy comparable to that of Zhang et al. [27], multiple independently initialized base learners (ELM or

WELM) were trained on bootstrap resamples of the training partition and combined via weighted majority voting over the discrete allele pair output, with each base learner's vote weighted by its validation-partition posterior probability. Because the base ELM's output weights depend on the random draw of input-to-hidden weights, individual models can vary somewhat from run to run; the ensemble is intended to reduce this prediction variance without materially increasing training time, since every base learner remains a single closed-form fit and the ensemble members can be trained in parallel.

### S3.4. Concordance with Confirmatory High-Resolution Typing

ELM top-1 concordance exceeded that of haplotype frequency imputation at all five loci, by 0.4–1.6 percentage points, and exceeded that of the equivalently sized MLP by 2.1–4.4 percentage points (Table S2). Gradient-boosted trees achieved the highest concordance at every locus, with the largest margins over the ELM at HLA-B and HLA-DRB1, the two most polymorphic loci, but at a higher training cost (Table S3). Top-3 concordance exceeded 98% for all loci and methods except the MLP, so the correct genotype was usually among the next most probable predictions when the top-1 call was incorrect.

**Table S2. Top-1 allele pair concordance** *with confirmatory high-resolution typing by method and locus (five-fold, ancestry-stratified cross-validation), simulated reference dataset.*

| Locus | Haplotype freq. imputation | MLP | Gradient-boosted trees | ELM (proposed) |
|---|---|---|---|---|
| HLA-A | 94.8% [94.5–95.1%] | 92.1% [91.7–92.5%] | 95.6% [95.3–95.9%] | 95.3% [95.0–95.6%] |
| HLA-B | 90.2% [89.7–90.6%] | 87.4% [86.9–87.9%] | 93.0% [92.6–93.4%] | 91.8% [91.4–92.2%] |
| HLA-C | 93.6% [93.2–94.0%] | 90.5% [90.0–91.0%] | 94.4% [94.0–94.8%] | 94.1% [93.7–94.5%] |
| HLA-DRB1 | 89.7% [89.3–90.1%] | 86.9% [86.4–87.4%] | 92.6% [92.2–93.0%] | 91.0% [90.6–91.4%] |
| HLA-DQB1 | 96.5% [96.2–96.8%] | 94.8% [94.5–95.1%] | 97.0% [96.7–97.2%] | 96.9% [96.6–97.1%] |

### S3.5. Performance in Under-represented Ancestry Groups

The advantage of the ELM over haplotype frequency imputation was largest in the ancestry categories with the fewest reference haplotypes, in which haplotype frequency imputation concordance fell by 6–11 percentage points relative to the best-represented category, against 3–5 percentage points for the ELM. This is consistent with a model that learns from genotype co-occurrence and is thus less dependent on the dense haplotype-level representation of each population, although it remains sensitive to sample size.

### S3.6. Computational Efficiency

Because ELM output weights were obtained in a single closed-form step, full training was roughly an order of magnitude faster than for the MLP and gradient-boosted trees, and two to three orders of magnitude faster than recomputing haplotype frequency tables (Table S3).

**Table S3. Wall-clock training time** *by method, simulated reference dataset. Absolute values are hardware-dependent; the ordering reflects the closed-form nature of ELM training.*

| Method | Training time (5 loci, full cohort) | Retraining cadence feasible |
|---|---|---|
| Haplotype frequency imputation | Several hours | Weekly/monthly batch |
| MLP (back-propagation) | 40-90 minutes | Daily batch |
| Gradient-boosted trees | 25-60 minutes | Daily batch |
| ELM (proposed) | 1–4 minutes | Same-day/on-demand |

### S3.7. Comparison of the Base ELM and Its Extensions

Among the ELM variants, KELM and Ensemble ELM exhibited small, consistent gains over the base ELM (0.2–1.4 percentage points per locus), and WELM performed on par with or marginally below it (Table S4). On alleles below the locus median frequency, WELM

showed the largest gain (68.7% versus 58.4% for the base ELM), at a small cost to the overall frequency-weighted accuracy; Ensemble ELM had by far the smallest run-to-run variability (SD 0.6 versus 2.1–2.6 percentage points; Table S5). KELM required the longest training time, because its kernel matrix scales quadratically with training-set size. The choice among variants should therefore follow the failure mode a registry most wants to avoid: WELM for equitable service across the allele spectrum, Ensemble ELM for reproducibility, and KELM where longer batch training is acceptable.

**Table S4. Top-1 concordance and training time by ELM variant***, simulated reference dataset.*

| Method | HLA-A | HLA-B | HLA-C | HLA-DRB1 | HLA-DQB1 | Training time (5 loci) |
|---|---|---|---|---|---|---|
| Base ELM | 95.3% [95.0–95.6%] | 91.8% [91.4–92.2%] | 94.1% [93.7–94.5%] | 91.0% [90.6–91.4%] | 96.9% [96.6–97.1%] | 1-4 min |
| KELM | 96.0% [95.7–96.3%] | 93.1% [92.7–93.5%] | 94.9% [94.5–95.2%] | 92.4% [92.0–92.8%] | 97.1% [96.8–97.3%] | 20-45 min |
| WELM | 95.0% [94.7–95.3%] | 91.2% [90.8–91.6%] | 93.8% [93.4–94.2%] | 90.6% [90.2–91.0%] | 96.7% [96.4–97.0%] | 1-4 min |
| Ensemble ELM (10 members) | 96.1% [95.8–96.4%] | 92.9% [92.5–93.3%] | 94.8% [94.4–95.1%] | 92.1% [91.7–92.5%] | 97.2% [96.9–97.4%] | 8-15 min |

**Table S5. Accuracy on alleles below the locus median frequency***, and standard deviation of top-1 concordance across ten random initializations, by ELM variant, simulated reference dataset. Confidence intervals are not given because the evaluable count of the rare-allele subset was not tracked per method.*

| Method | Rare-allele accuracy (below-median-frequency alleles) | Top-1 concordance, SD across 10 random initializations |
|---|---|---|
| Base ELM | 58.4% | ± 2.6 pp |
| KELM | 61.2% | ± 2.1 pp |
| WELM | 68.7% | ± 2.4 pp |
| Ensemble ELM (10 members) | 63.5% | ± 0.6 pp |

## Section S4. Additional Haplotype Frequency Comparators on the Greek Registry

This section reports, in full, the three haplotype frequency baselines summarized in the main text (Sections 2.6 and 3.3). The application of GRIMM to the external ORAM and GRPT cohorts is reported in the main text (Section 3.2, Table 10).

### S4.1. Two-Locus EM Haplotype Frequency Baseline

*Evaluable set: donors with unambiguous two-field confirmatory ground truth at both HLA-A and HLA-B simultaneously.*

We implemented a classical two-locus (HLA-A~HLA-B) haplotype frequency EM algorithm from first principles and ran it directly on this registry's data. This is a deliberately narrower comparator than a full production tool: it estimates a single A~B haplotype frequency distribution rather than a joint five-locus distribution, so it is not equivalent to running HaploStats, GRIMM, or Hapl-o-Mat, which model all five loci jointly and add features such as ambiguity code resolution and multi-population reference panels. We report it as a transparent, reproducible lower bound on EM-based performance, not as a substitute for benchmarking those tools directly.

During training, we used the standard genotype-based EM for haplotype frequencies (expectation over phase given unphased two-field HLA-A/HLA-B genotype pairs, closed-form maximization each iteration) on the subset of HTO donors with unambiguous two-field typing at both loci (n = 89,666; 28,449 distinct genotype combinations). The algorithm converged within 10 iterations (training

time under one second); the highest-frequency estimated haplotypes were well-established associations (e.g. A*01:01~B*08:01, A*02:01~B*51:01), a useful internal sanity check on the implementation (Figure S3).

We then evaluated a missing-locus imputation task analogous to Experiment 1 in the main text (Table 6, inferring HLA-B from HLA-A alone): for each donor in the verified donor-independent ORAM and GRPT cohorts with unambiguous two-field truth at both loci, the true HLA-A diplotype was supplied and the full HLA-B diplotype was predicted as the highest-posterior-probability pair under the estimated haplotype frequencies, and then compared with the confirmatory HLA-B typing. On ORAM (n = 2,305), the baseline returned a call for 53.9% of donors (1,243/2,305) with 4.7% top-1 accuracy among called donors (58/1,243); accuracy increased and the call rate fell sharply as the posterior-probability threshold increased, reaching 50.0% accuracy at Post-P >= 0.5, but for only 2 of 2,305 donors (Table S6, Figure S4). GRPT provided too few donors with unambiguous truth at both loci for a stable estimate (n = 6) and is not reported. The ELM framework substantially outperformed this baseline on the same task; instead of treating this as evidence that EM-based methods in general underperform compared to the ELM framework, we attribute this primarily to the EM baseline using only one conditioning locus versus the ELM's use of several simultaneously known loci, and secondarily to its narrower two-locus scope.

**Table S6. Two-locus (HLA-A to HLA-B) EM haplotype frequency baseline**, *missing-locus imputation task, evaluated on verified donor-independent ORAM donors (n=2,305) with unambiguous two-field truth at both loci.*

| Post-P threshold | Call rate | HLA-B accuracy (called) | n called/n total |
|---|---|---|---|
| none (all calls) | 53.9% [51.9–56.0%] | 4.7% [3.6–6.0%] | 1,243/2,305 |
| >= 0.3 | 0.5% [0.3–0.9%] | 33.3% [13.8–60.9%] | 12/2,305 |
| >= 0.5 | 0.1% [0.0–0.3%] | 50.0% [9.5–90.6%] | 2/2,305 |
| >= 0.7 | 0.1% [0.0–0.3%] | 50.0% [9.5–90.6%] | 2/2,305 |

## S4.2. GRIMM Applied to the HTO Training Population

We also ran GRIMM on the HTO training registry itself (117,345 donors), with the reference panel and configuration settings matching those used for the ORAM/GRPT runs (Section 3.2) and scored identically. The overall call rate was 30.6% (35,907/117,345). The per-locus first-field accuracy among called donors was 75.4-98.0% across the five loci, with joint accuracy (per-donor evaluable loci) of 72.0% (Table S7, Figure S7). This is directly comparable with the external validation presented in Section 3.2, where GRIMM's call accuracy was 74.9% jointly on ORAM and 97.4% jointly on GRPT. Note that 73.3% of HTO donors are typed at unambiguous two-field resolution at all five loci (Table 1), so the 30.6% call rate reflects GRIMM's reference panel matching rather than a lack of typing information in this cohort.

**Table S7. GRIMM performance on the HTO training population compared with the external ORAM and GRPT cohorts (Table 10), first-field top-1 concordance (Section S4.2). As in Table 10, joint accuracy is scored per donor over whichever loci that donor has both a GRIMM call and ground truth for, not a fixed five-locus requirement (Section 3.2).**

| Cohort | Call rate | Per-locus accuracy range | Joint accuracy | Status |
|---|---|---|---|---|
| HTO (training) | 30.6% (35,907/117,345) [30.34–30.86%] | 75.4–98.0% [endpoints' 95% CI: 75.0–75.8% to 97.9–98.1%] | 72.0% [71.5–72.5%] | Table 10 |
| ORAM (external) | 47.9% (9,358/19,535) | 80.3–99.9% [endpoints' | 74.9% [74.0–75.8%] | Table 10 |

| | | | | |
|---|---|---|---|---|
| | [47.2–48.6%] | 95% CI: 79.5–81.1% to 99.8–99.9%] | | |
| GRPT (external) | 1.8% (78/4,353) [1.4–2.2%] | 97.2–100% [endpoints' 95% CI: 91.0–99.3% to 95.2–100.0%] | 97.4% [91.0–99.3%] | Table 10 |

### S4.3. Open Source HaploStats-Inspired Baseline (hlaR ImputeHaplo)

*Evaluable set: donors with unambiguous two-field confirmatory ground truth, scored per locus; therefore, the per-locus n differs by locus and cohort and is reported explicitly below rather than assumed constant.*

NMDP's HaploStats [10] is a live, single-donor web service with no public batch-processing interface, so a like-for-like batch run analogous to the GRIMM comparison was not possible. As a partial substitute, we obtained five-locus output from the ImputeHaplo function of the open source R package hlaR [35], which performs haplotype frequency imputation using a reference table sourced from HaploStats. The package's own documentation cautions that its accuracy is lower than that of the HaploStats web service itself and that results should be used with caution; we therefore report it as an open source HaploStats-inspired baseline rather than as equivalent to the NMDP service.

The call rate on GRPT was 98.3% (4,278/4,353), which is far higher than GRIMM's 1.8% on the same cohort and consistent with haplotype frequency imputation being more tolerant of the ambiguous, low-resolution input that characterizes most GRPT donors (Table 4) than GRIMM's reference panel graph matching. However, because so few GRPT donors carry unambiguous two-field ground truth at any locus, the evaluable samples are very small: n = 2 (HLA-DQB1) to n = 64 (HLA-DRB1) per locus, with only n = 2 donors evaluable at all five loci simultaneously. Considering this caveat, the per-locus first-field accuracy among called donors ranged from 18.2% (HLA-C, n = 11) to 90.6% (HLA-DRB1, n = 64), and the joint accuracy was 0% on the n = 2 evaluable sample (Table S8, Figures S8-S9). These figures are illustrative of a much higher call rate at a markedly lower accuracy than GRIMM on the same cohort, not a statistically reliable estimate of hlaR ImputeHaplo's, or HaploStats', true accuracy.

On the verified donor-independent ORAM cohort, hlaR ImputeHaplo returned a call rate of 88.7% (17,320/19,535), with a per-locus first-field accuracy ranging from 28.63% to 93.65% (n = 2,480 HLA-A; 3,074 HLA-B; 1,483 HLA-C; 1,285 HLA-DQB1; 6,930 HLA-DRB1). On the HTO training registry, the call rate was 53.88% (63,227/117,345), with the per-locus first-field accuracy ranging from 26.30% to 89.53% (n = 48,130 HLA-A; 49,670 HLA-B; 47,132 HLA-C; 49,253 HLA-DQB1; 51,864 HLA-DRB1). Both sets of evaluable-donor counts are consistent with the typing-resolution proportions in Table 1. The joint accuracy and a single all-loci n were not computed for either run. The call rate and per-locus accuracy across the three cohorts are summarized in Figures S10 and S11.

**Table S8. Method comparison across GRPT, ORAM, and HTO: ELM vs. GRIMM (Section 3.2) vs. hlaR ImputeHaplo, an open source HaploStats-inspired baseline (Section S4.3), first-field top-1 concordance.** *The ELM's per-locus accuracy range is reported at first-field resolution (five per-locus models trained on HTO and evaluated on GRPT/ORAM, averaged over 25 repeated runs; Section 3.2), rather than as a two-field lower bound. The 98.1% call rate is a single overall figure reported once for the ELM framework as a whole (Section 3.1), not measured separately per cohort; it is repeated in each ELM row here only for ease of comparison against the cohort-specific comparator rows. The GRPT hlaR ImputeHaplo accuracy figures rest on very small evaluable samples (n=2-64 per locus) and should be treated as illustrative only; the joint accuracy and evaluable n were not*

*computed for the ORAM and HTO hlaR ImputeHaplo runs (only the call rate and per-locus accuracy range).*

| Method | Call rate | Per-locus accuracy range | Joint accuracy | evaluable n (joint) |
|---|---|---|---|---|
| ELM — GRPT | 98.1% (overall) | 85-93% | – | – |
| ELM — ORAM | 98.1% (overall) | 78-91% | – | – |
| ELM — HTO | 98.1% (overall) | 80-94% | – | – |
| GRIMM — GRPT (Section 3.2) | 1.8% (78/4,353) [1.4–2.2%] | 97.2–100% [endpoints' 95% CI: 91.0–99.3% to 95.2–100.0%] | 97.4% [91.0–99.3%] | 77 |
| hlaR ImputeHaplo — GRPT (Section S4.3) | 98.3% (4,278/4,353) [97.8–98.6%] | 18.2–90.6% [endpoints' 95% CI: 5.1–47.7% to 81.0–95.6%] | 0.0% [0.0–65.8%] | 2 |
| hlaR ImputeHaplo — ORAM (Section S4.3) | 88.7% (17,320/19,535) [88.2–89.1%] | 28.63–93.65% [endpoints' 95% CI: 27.06–30.25% to 93.05–94.20%] | n/a | n/a |
| hlaR ImputeHaplo — HTO (Section S4.3) | 53.88% (63,227/117,345) [53.60–54.17%] | 26.30–89.53% [endpoints' 95% CI: 25.92–26.69% to 89.26–89.79%] | n/a | n/a |

### S4.4. In-House Hapl-o-Mat-Style Five-Locus EM Baseline

*Evaluable set: as in Section S4.3, donors with unambiguous two-field confirmatory ground truth, scored per locus; for the joint figure, over the subset of loci sharing the largest common evaluable-donor set.*

At the time of writing, neither Hapl-o-Mat [12] nor the NMDP HaploStats web service offers a batch interface that we could execute in our environment. As a close approximation, we implemented the core EM algorithm that both tools use—population haplotype frequency estimation via expectation maximization over unphased multi-locus genotypes, followed by the Bayesian posterior scoring of candidate diplotypes (Algorithm S5)—directly in Python, and ran it end to end on this study's own HTO/ORAM/GRPT data, extending the two-locus baseline of Section S4.1 to all five loci. We refer to it as an in-house Hapl-o-Mat-style baseline, not as the Hapl-o-Mat software itself, since the implementation details of the published tool (its convergence criteria, population-weighting options and ambiguity-resolution logic) are not reproduced here. Hapl-o-Mat is open source and estimates frequencies from the user's own genotypes, so a direct run remains feasible and is identified in the main text (Section 5) as the appropriate next step.

During training, we used the standard genotype-based EM, extended from two to five loci, on the 86,002 HTO donors with unambiguous two-field typing at all five loci (72,336 distinct genotype combinations). The algorithm converged within 25 iterations (training time under two seconds on the Titanas server); the highest-frequency estimated five-locus haplotype was A*01:01~B*08:01~C*07:01~DQB1*02:01~DRB1*03:01, the well-known AH8.1 ancestral haplotype, and the second one was A*02:01~B*18:01~C*07:01~DQB1*03:01~DRB1*11:04—both biologically plausible for this population. Thus, this was a useful sanity check on the five-locus extension.

Unlike with the two-locus baseline, this evaluation had to handle GRPT's typing pattern directly: HLA-A and -B are populated for all 4,353 GRPT donors, HLA-DRB1 for 3,611 (83.0%), HLA-C for only 37, and HLA-DQB1 for only 2. Donors

were therefore scored on whichever loci they had both typing input and confirmatory two-field truth for, marginalizing over untyped loci by selecting, among haplotypes consistent with the typed loci, the pairing with the highest joint estimated frequency.

The call rate was 63.8% (2,777/4,353) on GRPT, 55.5% (10,847/19,535) on ORAM, and 48.00% (56,326/117,345) on HTO—intermediate in every case between GRIMM's and hlaR ImputeHaplo's call rate on the same cohort. On GRPT, the evaluable samples are very small at every locus (n = 2 HLA-A; 4 HLA-B; 4 HLA-C; 45 HLA-DRB1; no evaluable call at HLA-DQB1); thus, the resulting 100.0% figures are illustrative only (Figures S12-S13). On HTO, the per-locus first-field accuracy ranges from 90.74 to 97.52%—n/k by locus: 42,185/38,277 (HLA-A, 90.74%), 43,174/40,976 (HLA-B, 94.91%), 41,189/38,452 (HLA-C, 93.36%), 42,738/40,542 (HLA-DQB1, 94.86%), 47,458/46,280 (HLA-DRB1, 97.52%)—with a joint five-loci accuracy of 90.44% (37,101/41,023). On ORAM, the per-locus accuracy ranges from 26.25-92.45% (n = 1,558 HLA-A; 2,076 HLA-B; 1,023 HLA-C; 916 HLA-DQB1; 4,387 HLA-DRB1; HLA-DQB1 k = 361, 39.41%), with a joint accuracy of 6.46% (n = 712, all five loci) or 6.07% (n = 890, four loci). The contrast between 90.44% on HTO and 6.46% on ORAM reflects ORAM's markedly lower and more variable per-locus accuracy rather than a scoring artefact; note that HTO is the population from which this baseline's frequencies were estimated, so its HTO figures are in-sample. All three comparators are summarized together in Table 11, with the call rate and accuracy presented by cohort in Figures S14 and S15.

### S4.5. Accuracy Stratified by Allele Vocabulary Membership

**Table S9. Accuracy stratified by allele vocabulary membership, per locus, and cohort.** A donor counts as in-vocabulary at a locus when both of that donor's confirmatory alleles belong to the 99%-coverage vocabulary derived from the HTO training registry (Tables 4 and 5); counts are per donor, so the out-of-vocabulary share here exceeds the per-call share in Table 4. The two-field accuracy in the out-of-vocabulary stratum is zero by construction, since the model cannot represent an allele absent from its output vocabulary, but the first-field accuracy in that stratum is not, because a call truncated to the first field can still be correct. The Total column reproduces the per-locus first-field accuracy reported in Section 3.2. The denominators at HLA-DQB1 in GRPT (N=4) and at HLA-A, -B, and -C in GRPT (N=15-27) are very small, and their rows are presented for illustrative purposes only.

| **Cohort** | **Locus** | **Donors (N)** | **In-vocabulary n** | **Accuracy in-vocab. 1F (k/n)** | **Accuracy in-vocab. 2F (k/n)** | **Out-of-vocabulary n** | **Accuracy out-of-vocab. 1F (k/n)** | **Accuracy out-of-vocab. 2F** | **Total 1F (k/N)** |
|---|---|---|---|---|---|---|---|---|---|
| ORAM | HLA-A | 16,758 | 6,073 | 89.7% (5447/6073) | 89.7% (5447/6073) | 10,685 | 71.6% (7650/10685) | 0% by construction | 78.2% (13097/16758) |
| ORAM | HLA-B | 17,027 | 8,851 | 94.5% (8364/8851) | 93.6% (8285/8851) | 8,176 | 73.2% (5985/8176) | 0% by construction | 84.3% (14349/17027) |
| ORAM | HLA-C | 8,369 | 2,395 | 94.0% (2251/2395) | 94.0% (2251/2395) | 5,974 | 74.9% (4475/5974) | 0% by construction | 80.4% (6726/8369) |
| ORAM | HLA-DRB1 | 18,827 | 10,846 | 96.1% (10423/10846) | 93.6% (10152/10846) | 7,981 | 75.3% (6010/7981) | 0% by construction | 87.3% (16433/18827) |
| ORAM | HLA-DQB1 | 3,200 | 2,162 | 96.6% (2088/2162) | 93.5% (2021/2162) | 1,038 | 78.9% (819/1038) | 0% by construction | 90.8% (2907/3200) |
| GRPT | HLA-A | 15 | 10 | 90.0% (9/10) | 90.0% (9/10) | 5 | 80.0% (4/5) | 0% by construction | 86.7% (13/15) |
| GRPT | HLA-B | 21 | 15 | 93.3% (14/15) | 93.3% (14/15) | 6 | 83.3% (5/6) | 0% by construction | 90.5% (19/21) |
| GRPT | HLA-C | 27 | 16 | 93.8% (15/16) | 93.8% (15/16) | 11 | 72.7% (8/11) | 0% by construction | 85.2% (23/27) |
| GRPT | HLA-DRB1 | 165 | 122 | 96.7% (118/122) | 94.3% (115/122) | 43 | 81.4% (35/43) | 0% by construction | 92.7% (153/165) |
| GRPT | HLA-DQB1 | 4 | 4 | 100.0% (4/4) | 75.0% (3/4) | 0 | n/a | n/a | 100.0% (4/4) |

| Cohort | Locus | Donors (N) | In-vocabulary n | Accuracy in-vocab. 1F (k/n) | Accuracy in-vocab. 2F (k/n) | Out-of-vocabulary n | Accuracy out-of-vocab. 1F (k/n) | Accuracy out-of-vocab. 2F | Total 1F (k/N) |
|---|---|---|---|---|---|---|---|---|---|
| ORAM (all loci) | Pooled | 64,181 | 30,327 | 94.2% (28,573/30,327) | 92.8% (28,156/30,327) | 33,854 | 73.7% (24,939/33,854) | 0% by construction | 83.4% (53,512/64,181) |
| GRPT (all loci) | Pooled | 232 | 167 | 95.8% (160/167) | 93.4% (156/167) | 65 | 80.0% (52/65) | 0% by construction | 91.4% (212/232) |
